# Sustainable Approaches to Corrosion Protection in Reinforced Concrete: A Critical Review of Green Inhibitors, Surface Treatments, Self-Healing Systems, Nanomaterials, Smart Coatings, and Life-Cycle-Based Design

**DOI:** 10.3390/ma19204345

**Published:** 2026-10-15

**Authors:** Md Arifuzzaman, Ziad Nayef Shatnawi, Abdullah Al Mamun, Ayed Eid Alluqmani

**Affiliations:** 1 Department of Civil and Environmental Engineering, College of Engineering, King Faisal University, Al-Ahsa 31982, Saudi Arabia; 2 Lyles School of Civil and Construction Engineering, Purdue University, West Lafayette, IN 47907, USA; 3 Department of Civil Engineering, Faculty of Engineering, Islamic University of Madinah, Al-Madinah Al-Munawara, Prince Naif Ibn Abdulaziz, Al Jamiah, Medina 42351, Saudi Arabia

**Keywords:** reinforced concrete, chloride-induced corrosion, carbonation, green corrosion inhibitors, surface treatment, self-healing concrete, smart coatings, supplementary cementitious materials, service life, life cycle assessment, machine learning

## Abstract

Corrosion of steel reinforcement is still the main reason reinforced concrete structures fail before the end of their planned service life. Every early repair costs money, and it also adds new cement, steel, and emissions. This review examines protection methods that can extend service life while lowering environmental impact. It covers green and nano-enabled corrosion inhibitors, hydrophobic and polymer-modified surface treatments, autogenous, bacterial, and capsule-based self-healing systems, nanomaterials, and stimulus-responsive smart coatings. It also covers supplementary cementitious materials, limestone calcined clay cement, and alkali-activated binders as the first barrier against chlorides and carbon dioxide. The review then links these materials to durability-based design, probabilistic service-life models, life cycle assessment, machine learning, and sensor-based monitoring. Three findings stand out. First, most green inhibitors and smart coatings have been tested in pore solution or in short accelerated tests, and long-term data in real concrete are scarce. Second, a gain in chloride resistance only counts as a sustainability gain when it is expressed per year of service life, including maintenance. Third, data-driven models predict chloride transport well inside their training data, but few of them respect physical limits or report uncertainty. We grade each method by the evidence behind it, from pore-solution tests to long-term field use, and by its technology maturity. Most of the newer methods have not yet left the laboratory. A worked example shows that holding the chloride diffusion coefficient at its 28-day value understated the time to corrosion initiation of fly ash and slag concretes by a factor of about 4 to 7. Here, sustainability refers mainly to climate impact, expressed as global warming potential per year of service life. We propose a design loop with two decision gates. Unlike existing service-life or life cycle frameworks, it checks reliability and climate impact together, and it gives credit only to methods with measured inputs in mortar or concrete. It connects binder choice, added protection, probabilistic service-life prediction, and life cycle metrics, and it updates itself with monitoring data. The review closes with a ranked list of research gaps and practical steps for researchers, designers, and asset owners.

## 1. Introduction

Reinforced concrete carries most of the world’s bridges, ports, tunnels, and buildings. Its long life depends on a thin passive film that forms on the steel in the highly alkaline pore solution of concrete [1,2]. When chloride ions reach the steel in a high enough amount, or when carbonation lowers the pH, this film breaks down and corrosion starts [3,4,5]. The rust takes up more space than the steel it replaces. It cracks the cover, weakens the bond between steel and concrete, and reduces load capacity in bending and shear [6,7,8].

The cost of corrosion is not only financial. Each early repair brings new cement, new steel, traffic delays, and more emissions. Angst has argued that steel corrosion may be the weakest link for low-carbon concrete, because lowering the clinker content changes the alkaline buffer that protects the steel [9]. This creates a real tension. Designers want less clinker to cut carbon, but they also need the structure to last. A mix with a slightly higher initial footprint can still be the greener option if it doubles the service life [10,11]. We think this point is often missed when new materials are called “sustainable” based on their mix design alone.

Protection methods have moved well past calcium nitrite and epoxy-coated bars. Researchers now test plant extracts as inhibitors, silane and lithium-based surface treatments, bacteria that seal cracks, nanoparticles that refine the pore structure, and coatings that release inhibitors only when corrosion begins [12,13,14,15,16]. Design practice has also changed. Codes and researchers are moving from fixed cover and water-to-binder rules toward performance-based and probabilistic service-life design [11,17,18]. Machine learning now appears in most durability journals, often to predict chloride diffusion from mix proportions [19,20,21].

Several good reviews already exist. They cover green inhibitors [12,22,23], concrete surface treatments [24], self-healing concrete [25,26], corrosion assessment and control [27], and durable concrete in chloride environments [28]. Most of them, however, look at one family of methods at a time. What is still missing, in our view, is a review that puts the protective measure, the service-life model, and the life cycle metric in the same frame. A green inhibitor that works for five years in the field is not green if the structure needs major repair at year twenty.

**Figure 1:**
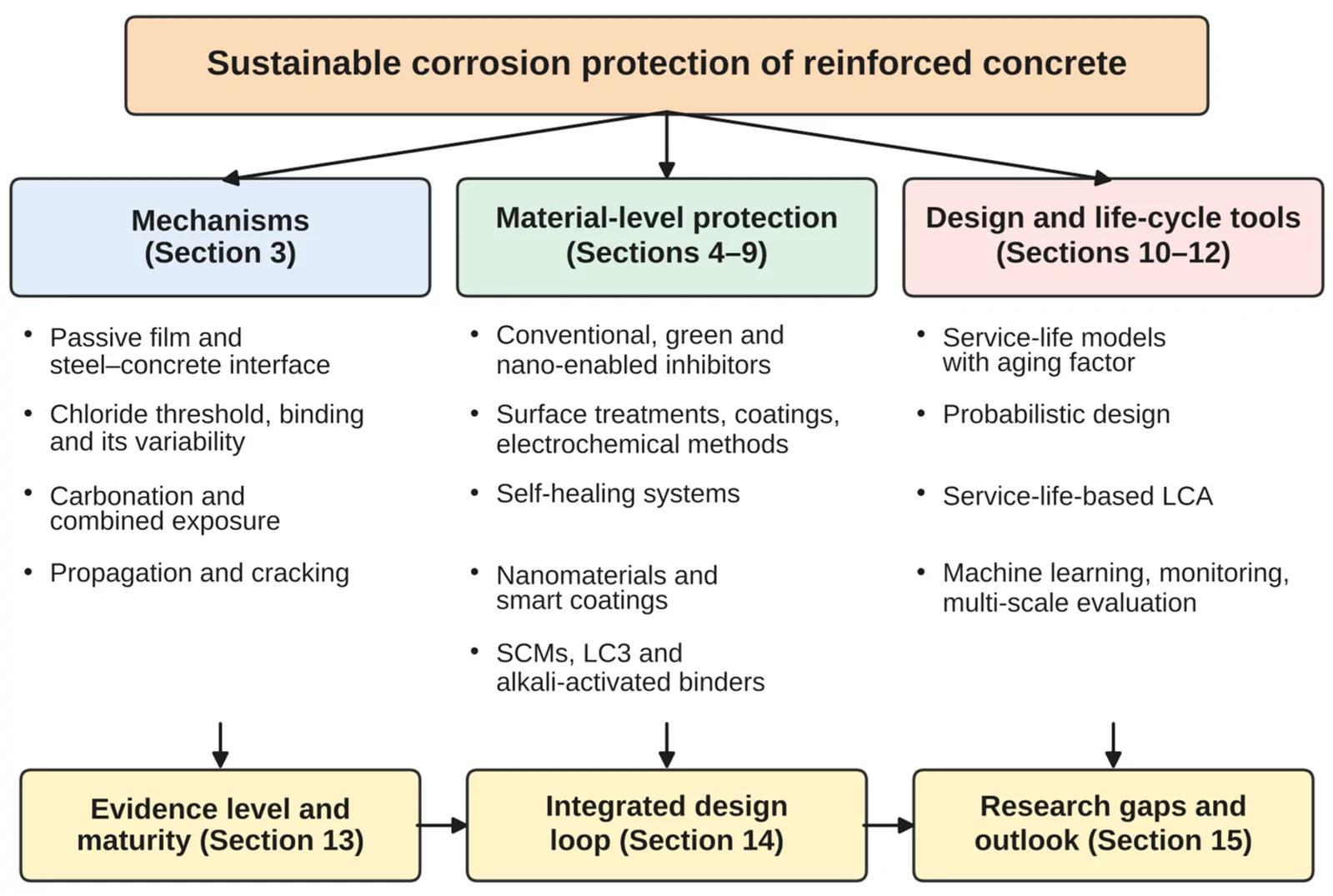
Structure of this review. Corrosion mechanisms (Section 3) set the targets. Material-level protection methods (Section 4, Section 5, Section 6, Section 7, Section 8 and Section 9) act on those targets. Design and life-cycle tools (Section 10, Section 11 and Section 12) decide whether a method is truly sustainable. Section 13 grades the evidence and maturity of each method, and Section 14 and Section 15 bring the parts together.

This review tries to close that gap. It has five aims: (i) explain the corrosion processes that each protection method targets; (ii) assess the evidence for eco-friendly inhibitors, surface treatments, self-healing systems, nanomaterials, and smart coatings; (iii) discuss supplementary cementitious materials (SCMs) and low-carbon binders as the first line of defense; (iv) connect these materials to durability-based design, life cycle assessment (LCA), predictive modeling, and multi-scale evaluation; and (v) identify research gaps. Figure 1 shows how the review is organized.

Two points of scope need stating at the outset. First, we use the word “sustainable” in a narrow sense. Our main indicator is environmental: the global warming potential (GWP) per year of service life, including maintenance. Cost, resource use, and toxicity enter the discussion where the source studies report them, but we do not score social or economic aspects. Second, this review tries to do more than summarize recent papers. It adds three things. Firstly, it grades each protection method by the evidence behind it, from pore-solution tests to long-term field use, and by its technology maturity (Section 13). Secondly, it translates reported effects into the inputs that service-life and LCA models need, such as the diffusion coefficient, its aging factor, the chloride threshold, and the reapplication interval (Section 10 and Section 13). Thirdly, it proposes a design loop with explicit decision gates that a protection method must pass before it is called sustainable (Section 14). The parts of this loop already exist in other frameworks. What we add is the way they are joined, and Section 14 compares the loop with earlier durability and sustainability frameworks to show where it differs and what it changes in practice.

## 2. Review Approach

This is a critical narrative review, not a systematic review. We still followed a defined search and selection procedure so that readers can judge its coverage. We searched Scopus, Web of Science Core Collection, and Google Scholar, and we added backward and forward citation tracking from recent reviews [12,22,24,25,28]. Search strings combined one corrosion term (“steel corrosion”, “reinforcement corrosion”, “chloride”, or “carbonation”) with “reinforced concrete” and with one method term (“corrosion inhibitor”, “green inhibitor”, “surface treatment”, “self-healing”, “nanomaterial”, “smart coating”, “supplementary cementitious material”, “service life”, “life cycle assessment”, or “machine learning”). We limited the search to sources in English. Most sources date from 2010 to 2026. We kept older sources when they were standard references for a mechanism or a test method.

We screened titles and abstracts first and then read the full texts. A source was included when it met three conditions: (i) it was a peer-reviewed journal paper, a RILEM state-of-the-art report, a standard reference book, a standard, or a design guideline; (ii) it dealt with steel in cement-based materials, or with a mechanism that also controls steel in concrete; and (iii) it reported a result that can be checked, such as a test method, a measured property, or a model with stated inputs. We excluded conference abstracts and reports without peer review. Studies on bare steel in acid or neutral saline media were excluded unless they explained a mechanism we needed, and we say so in the text where we use them. The final set has 119 sources published between 1993 and 2026. A few sources come from neighboring fields: metal oxidation, machining, soil stabilization, and structural control. We cite them only for a mechanism or a method that carries over to steel in concrete, and we state the link each time we use one.

We read each study with three questions in mind. Where does the method act: on transport, on the chemistry at the steel surface, on the steel itself, or on cracks? How long was the test, and was it done in solution, mortar, concrete, or a real structure? Did the authors report anything that can feed a service-life or life cycle model, such as a diffusion coefficient, a chloride threshold, or a corrosion rate? These questions shape the tables and the critical comments in each section.

We also gave each study an evidence level based on its test system: L1, pore solution or other liquid media; L2, mortar or concrete specimens in accelerated laboratory tests; L3, mortar or concrete in long-term laboratory or natural exposure (one year or more); L4, pilot or field application; and L5, field performance documented over ten years or more. Section 13 uses these levels to rank the methods. To keep laboratory findings apart from field-proven practice, each method section (Section 4, Section 5, Section 6, Section 7, Section 8, Section 9 and Section 12) ends with a short maturity note. The note gives the highest evidence level we found, the indicative technology readiness level (TRL) from Table 8, and one of three readiness groups: A, ready for routine design; B, pilot or early commercial use that still needs monitoring; and C, laboratory stage only. Section 13.3 brings these notes together in one synthesis table (Table 9).

## 3. Corrosion of Steel in Concrete: What Protection Must Address

### 3.1. Passivity and the Steel–Concrete Interface

Fresh concrete has a pore solution pH of roughly 12.5 to 13.5. At this pH, carbon steel forms a thin, protective oxide film [1]. The film is not perfect, and the zone around the bar is not uniform. Mill scale, rust from storage, air voids, and bleed water under horizontal bars all change the local conditions [29,30]. RILEM TC 262-SCI reviewed how these local features affect the start of chloride-induced corrosion. The committee showed that several of these effects are still unclear and that test results often conflict [31]. Steel surface condition alone can shift the onset of corrosion, as Boubitsas and Tang found when they compared bars with different surface states [32]. For protection design, the lesson is simple. Any method that improves or stabilizes the interface, such as a good inhibitor or a dense, well-cured cover, has value beyond what bulk tests show.

The passive film is also not static. It dissolves and re-forms all the time, and corrosion starts where local breakdown outpaces repair [1,3]. Work on other alloys in alkaline media shows how fine this balance can be. Li et al. removed the oxide film from Alloy 690 in alkaline hydrogen peroxide and traced the process to a cycle of selective dissolution and repassivation [33]. That study served nuclear decontamination, not concrete. It still supports a point that matters for protection design. An inhibitor or a surface treatment does the most good when it shifts this balance toward repair at the steel surface, and not only when it slows chloride on its way through the cover.

### 3.2. Chloride-Induced Corrosion

Chlorides come from seawater, de-icing salts, or contaminated mix materials. They move into concrete by diffusion, capillary suction, and permeation, and wet–dry cycles speed up the process near the surface [4,28]. Part of the chloride binds to the cement paste. Aluminate phases can form Friedel’s salt, and calcium silicate hydrate adsorbs chloride on its surface. Only free chloride drives corrosion, so binding lowers the risk [34,35].

Corrosion starts when the chloride content at the steel passes a critical value, often called the chloride threshold. This value is not a material constant. A widely cited review found a very wide spread of reported thresholds, caused by different test methods, steel surfaces, binders, and exposure conditions [3]. Researchers keep proposing better ways to measure it. Examples include potential monitoring during slow chloride uptake in slag concretes [36], a short-term test for systems with corrosion-inhibiting admixtures [37], and regression models that link the threshold to the water-to-binder ratio and to the fly ash and slag contents [38,39]. We see the threshold as one of the largest sources of uncertainty in chloride service-life models, and it deserves more attention than it gets in green-material papers.

The spread in reported thresholds is large. Angst et al. collected values from about 0.02% to about 3% total chloride by binder mass, a range of more than two orders of magnitude [3]. Several factors explain this scatter. The steel–concrete interface comes first. Air voids, bleed-water gaps, and mill scale at the bar lower the threshold, while a dense, lime-rich layer raises it [29,31,32]. The pore solution pH matters because chloride competes with hydroxide ions at the passive film. Binders that lower the hydroxide content can lower the threshold even when they bind more chloride, which is one reason why results for slag and fly ash concretes do not agree [3,36]. A lower water-to-binder ratio tends to raise the threshold [38,39]. Moisture and oxygen supply change the steel potential, and steel at more negative potentials, as in water-saturated concrete, tolerates more chloride [3]. Finally, the test method shifts the result. Electrically accelerated tests, admixed chloride, and natural diffusion give different values, and accelerated tests can mislead for inhibitors that carry an electric charge [37].

Reporting practice adds more scatter. Some studies give total chloride and others free chloride. Some normalize by binder mass and others by concrete mass. Values cannot be compared until they are put on the same basis. Design codes handle the scatter by treating the threshold as a random variable. The fib Model Code for Service Life Design, for example, uses a beta distribution with a mean of about 0.6% by binder mass for uncoated carbon steel [40]. The effect on predicted life is large. With the initiation-time solution given later in Equation (2), Cs = 3.0%, D = 0.5 × 10^−12^ m^2^/s, and a 50 mm cover, a threshold of 0.2% gives an initiation time of 23.6 years, 0.4% gives 35.2 years, 0.6% gives 48.2 years, and 1.0% gives 84.6 years. A change in Ccrit within the range seen in the literature changes the predicted life more than most protection methods do. For this reason, we ask that any study on a new protection method report its effect on the threshold, measured with embedded steel, and not only its effect on transport.

Exposure zone matters as much as the material. In the tidal and splash zones of marine structures, repeated wetting and drying moves chloride inward by capillary suction and then leaves it behind as the water evaporates. This creates a surface “convection zone” where Fick’s law does not hold, and the depth of this zone grows when load-induced microcracks are present [28]. Temperature and relative humidity also change the rate of transport, so the same concrete can have very different service lives in a hot, humid coast and in a cool, dry inland site [41]. For protection design, this means that surface treatments and self-healing agents are most useful exactly where the convection zone is deepest, while SCMs and low water-to-binder ratios govern the slower diffusion deeper in the cover [4,28].

### 3.3. Carbonation-Induced Corrosion

Carbon dioxide from the air reacts with calcium hydroxide and other hydrates. This lowers the pH near the steel to about 9, and the passive film is lost over a wide area [1]. The corrosion rate then depends mostly on moisture and oxygen. Sánchez Montero and co-workers followed carbonation-induced corrosion in real elements. They found that the average corrosion rate in elements with unacceptable section loss was about eight times higher than in cracked elements without section loss [42]. This means that the propagation phase is not steady, and a single corrosion rate in a model can mislead.

Chloride and carbonation often act together, for example in coastal cities or in car parks. Carbonation can release bound chloride and raise the free chloride at the steel [5,28]. Jiang et al. compared macrocell corrosion under carbonation, admixed chloride, and chloride penetration. At the same anodic potential drop, the macrocell current was higher for chloride-induced corrosion than for carbonation. Concretes with slag and fly ash were less affected by chloride contamination than plain Portland cement concrete, most likely because they bind more chloride [43].

The protection methods in this review do not work equally well against carbonation. Most of them were designed and tested for chloride exposure, so their value under carbonation needs a separate check. SCMs are the clearest case. They lower chloride diffusion, but they consume portlandite, so the carbonation front can move faster than in Portland cement concrete of the same strength [9,44]. LC3 shows the same trade-off [10,45]. Under carbonation, the designer has to rely on a lower water-to-binder ratio, a thicker cover, and longer curing rather than on the binder chemistry alone.

Surface treatments act in two ways. Film-forming coatings with low CO_2_ permeability slow carbonation directly, and repair practice uses them for this purpose [1,24,46]. Hydrophobic impregnations such as silanes do not block CO_2_. A drier cover may even carbonate a little faster, because CO_2_ diffuses more easily through air-filled pores. Their benefit under carbonation comes later, in the propagation phase. Once the steel is depassivated, the corrosion rate depends mainly on moisture, and a drier cover keeps that rate low [1]. Inhibitors have been studied far less under carbonation than under chloride attack, and the evidence for both admixed and migrating types is thin and mixed [1,47]. Electrochemical realkalization is the one method developed for carbonated concrete, and it has a long field record [1,27]. For self-healing, the open question is whether a healed crack restores alkalinity at the bar. Calcium carbonate in the crack seals the path, but it does not raise the pH of concrete that has already carbonated around the steel. Table 8 in Section 13 summarizes how each method performs under carbonation.

### 3.4. Propagation, Cracking, and Structural Effects

Once corrosion starts, the rate depends on concrete resistivity, moisture, oxygen supply, temperature, and the ratio of anode to cathode area. Otieno et al. reviewed corrosion-rate models and showed that many are hard to transfer between laboratory and field conditions [48]. Cracks make things worse. They act as fast paths for water and chloride, and numerical models built from tests on cracked and coated concrete show strong local attack at the crack [49,50]. At the member level, corrosion reduces flexural capacity [6,7] and shear capacity when stirrups corrode [8]. Fibers can change both corrosion resistance and bond behavior [51]. Bond needs its own model. Long et al. described the bond stress–slip law of steel in concrete with a bilinear cohesive model in finite element analysis. They then trained a deep learning model, a nonlinear autoregressive network with exogenous inputs (NARX), on the simulated responses. The network matched the finite element results with a correlation coefficient of 0.97, and a higher loading rate raised bond strength without changing the shape of the curve [52]. Their bars were not corroded, but the cohesive parameters give a clear place to enter corrosion damage in future work. Once capacity is lost, strengthening can restore it without demolition. Huang et al. modeled RC columns strengthened with an HPFL-BSP system under combined compression, bending, and torsion. A higher axial compression ratio raised the bearing capacity but reduced ductility, and a higher torsion-to-bending ratio lowered both capacity and stiffness [53]. Bridge piers and frame columns often carry such combined loads. In a life cycle view, strengthening is one more maintenance option with its own footprint, and it belongs in the maintenance term of Equation (5).

### 3.5. What This Means for Protection

**Figure 2:**
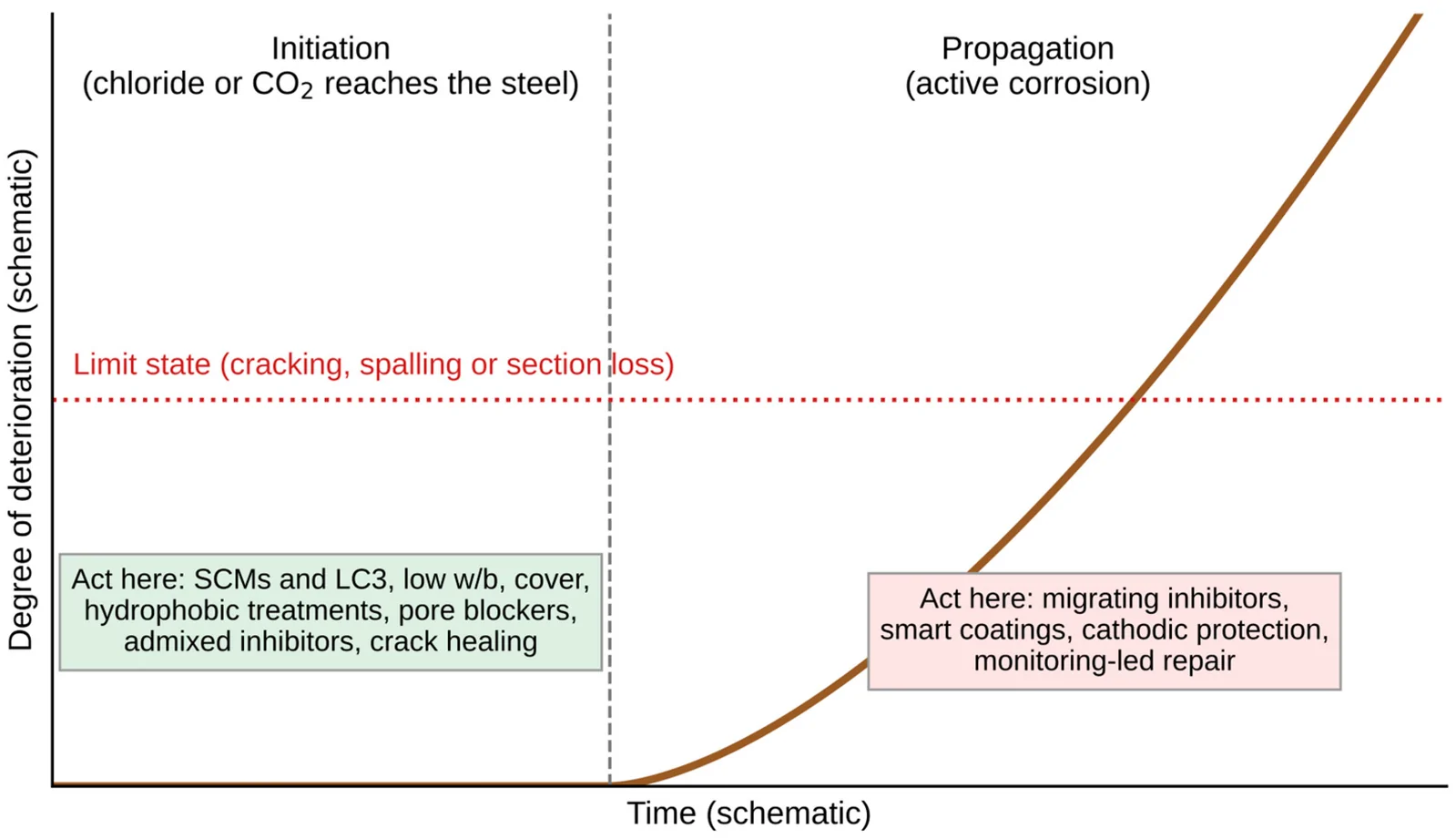
Schematic two-stage model of corrosion damage in reinforced concrete, showing where sustainable protection strategies act. The curve is qualitative; it is adapted from the two-stage concept described in [1].

Service life is usually split into an initiation phase and a propagation phase (Figure 2) [1,17]. Most sustainable strategies try to lengthen initiation. They slow chloride or carbon dioxide transport, raise the threshold, or seal cracks. A smaller group works during propagation. These include migrating inhibitors, smart coatings that respond to a pH or potential change, and repair guided by monitoring. Table 1 summarizes where each family of methods acts and what limits it.

## 4. Corrosion Inhibitors

### 4.1. Conventional Inorganic and Organic Inhibitors

Corrosion inhibitors are chemicals that slow corrosion when present at a small dose. In concrete, they are either mixed into fresh concrete or applied to the surface of existing structures so that they migrate toward the steel [27,47]. They are grouped by the reaction they block: anodic, cathodic, or mixed. Calcium nitrite is the classic anodic inhibitor and the usual benchmark. It helps oxidize ferrous ions and repair the passive film. Its main drawbacks are toxicity concerns and the need to keep a high nitrite-to-chloride ratio. At too low a dose, an anodic inhibitor can make local attack worse [12,28].

Organic inhibitors work mostly by adsorbing on the steel and forming a film that blocks charge transfer or water access. Ormellese et al. screened 80 organic compounds in synthetic pore solution. They then carried the nine best substances, mainly amines, aminoalcohols, carboxylates, and amino acids, into long-term tests in concrete [56]. The same group later used atomistic simulations to study how a triethylenetetramine-based inhibitor film sits on the passive layer and competes with chloride [65]. This kind of molecular work is useful because it explains why some molecules survive in pore solution and others do not.

Migrating corrosion inhibitors are attractive for existing structures because they need no demolition. Their weak point is proof of arrival. Several reviews point out that it is hard to confirm how much inhibitor reaches the steel and how long it stays there [22,47]. Test methods matter here as well. Karuppanasamy and Pillai showed that some accelerated chloride tests are not suitable for systems with anion-containing inhibitors, and they proposed a modified short-term threshold test [37].

Maturity note. Calcium nitrite has decades of field use and a product standard (ASTM C1582 [66]); it sits at evidence level L5, TRL 9, group A. Migrating inhibitors are in field use (L4, TRL 6–8, group B), but proof that enough inhibitor reaches the steel is still weak. For organic admixed inhibitors, long-term tests in concrete exist for a small number of substances [56].

### 4.2. Green Inhibitors from Plants and Wastes

Green inhibitors are made from plant extracts, food waste, or other natural sources. Their molecules usually contain nitrogen, oxygen, sulfur, or phosphorus, along with aromatic rings or double bonds. These features let them adsorb on the steel and form a protective layer [12,23]. Table 2 lists studies that tested such extracts with steel in concrete or in conditions close to it.

The results so far come from laboratory specimens (evidence level L2). Bambusa arundinacea leaf extract reduced corrosion of steel in concrete [67]. Olive leaf extract gave protection in concrete contaminated with seawater [70]. Maize gluten meal extract was studied with both electrochemical tests and theoretical calculations, which helped explain how its molecules bind to steel [71]. Neem (Azadirachta indica) leaf extract was compared with three commercial inorganic inhibitors under a chloride environment, and it did not change the fresh or hardened properties of the concrete in that study [72]. Other work combined plant extracts with silver nanoparticles [68] or used eco-addition inhibitors in reinforced concrete [69]. Only the first six studies in Table 2 tested steel inside a cement matrix. The last two used liquid media and serve here only to explain mechanisms.

We still urge caution. Casanova et al. compared green inhibitors with nitrite and separated accelerated tests from long-term tests. They found that the evidence from long-term tests is much thinner [12]. Many studies with very high inhibition efficiencies were done in acid solution, such as fenugreek seed extract for steel in phosphoric acid [73]. Those numbers do not transfer to pH 13. Few papers report chemical stability of the extract in pore solution over years. Zomorodian and Behnood proposed selection criteria that weigh efficiency, toxicity, cost, and compatibility with concrete [22]. We support that approach and suggest adding one more criterion: the inhibitor’s effect on the chloride threshold measured in mortar or concrete, not only its efficiency in solution.

Natural extracts also vary from batch to batch. The same plant species can give extracts with different amounts of active compounds, such as phenolics, alkaloids, and tannins. The amounts depend on where the plant grew, when it was harvested, which part was used, and which solvent and temperature were used for extraction [12,22,23]. Few papers test more than one batch, so a reported efficiency may describe one batch rather than the material. This matters in practice. A commercial admixture comes with a specified composition and a tolerance band, and a green inhibitor needs the same before a specifier can accept it. We suggest that studies report the plant origin and harvest season, the extraction protocol, the extract yield, and a chemical fingerprint, such as an FTIR or HPLC profile or the total phenolic content. They should also test at least three independent batches. Supply deserves thought as well. An extract that competes with food or feed, or that comes from a seasonal crop, may not be available in the volumes that concrete production needs.

Some extracts contain sugars or polysaccharides, which are known retarders of cement hydration. The papers we reviewed rarely quantify this effect, yet a practical benchmark already exists. ASTM C494 requires a normal (Type A) water-reducing admixture to keep the initial and final setting times within 1 h earlier and 1.5 h later than those of a control mix [75]. A green inhibitor that is not meant to act as a retarder should meet the same limit at its working dose. We recommend measuring setting time on concrete by penetration resistance (ASTM C403 [76]) or on mortar according to EN 480-2 [77]. The test should cover the intended dose and about twice that dose, to account for overdosing on site. If an extract retards setting beyond this range, the supplier should declare it, and the mix design should treat it like any retarding admixture.

Maturity note. Green inhibitors have reached evidence level L2 at best, that is, short laboratory tests with steel in mortar or concrete. We found no pilot or field use and no standard. Their indicative TRL is 3–4 (group C). The positive results in Table 2 describe laboratory behavior. They are not proof of performance in real structures.

### 4.3. Encapsulated and Nano-Enabled Inhibitors

A free inhibitor can leach out, react with cement phases, or be used up before corrosion starts. Encapsulation tries to fix this. The inhibitor is loaded into micro- or nanocontainers, such as layered double hydroxides, halloysite tubes, or polymer capsules, and it is released when a trigger appears, such as a drop in pH or the arrival of chloride [16,28,60]. Koleva showed that tailored polymeric nanomaterials can change the corrosion products and the protective layer on steel in cement-based systems, which points to self-healing of the steel surface itself [78]. The concept is sound, but it has not left the laboratory. However, almost all data come from laboratory specimens, and dosage, cost, and effects on hydration need more study before field use.

Maturity note. Encapsulated and nano-enabled inhibitors are at L1–L2 and TRL 2–4 (group C). We found no study in reinforced concrete under natural exposure.

### 4.4. Testing, Dosage, and Interaction with Binders

The way an inhibitor is tested decides what its result means. Tests in synthetic pore solution are fast and good for screening, but they leave out the cement paste, the steel–concrete interface, and the slow transport of chloride through the cover [12,56]. Tests in mortar or concrete are slower but closer to practice. Accelerated tests that drive chloride with an electric field can distort the result for inhibitors that carry an electric charge, which is why modified threshold tests have been proposed [37]. We recommend that any new inhibitor be reported with at least three results: its efficiency in pore solution, its effect on the chloride threshold in mortar or concrete, and its effect on setting time and 28-day strength.

Inhibitors and SCMs are often used together, and they can help each other. The SCM slows chloride ingress, so the inhibitor is used up more slowly, and the inhibitor adds a chemical barrier where the SCM is weak, for example under carbonation. There can also be conflict. Alumina-rich SCMs such as metakaolin may adsorb part of an organic inhibitor and reduce the amount that reaches the steel [28]. Very few studies test inhibitor type and binder type in a full factorial design. Until they do, designers cannot assume that the benefits add up [22,47].

## 5. Surface Treatments and Coatings

### 5.1. Concrete Surface Treatments

Surface treatments protect the cover concrete, which is the zone that decides how fast chlorides and carbon dioxide reach the steel. They fall into three groups: film-forming coatings, pore blockers, and hydrophobic impregnations such as silanes and siloxanes [24,46]. External coatings designed for both concrete and steel protection were studied as early as the 1990s [79].

Silane-based treatments are the best documented. Coppola et al. studied a silane-based surface-applied corrosion inhibitor. It cut the non-steady-state migration coefficient by 30% to 60% in accelerated tests. In natural diffusion tests, the apparent diffusion coefficient fell by about 75%. Resistivity data showed that the main mechanism was water repellence [13]. In a marine setting, surface treatments changed both the chloride diffusion coefficient and the capillary absorption of the cover [80]. Silane can also be added to the mix as a hydrophobic admixture, and this also affects steel corrosion [81]. Newer lithium-based and nano-modified waterproof coatings on mortar have shown resistance to salt attack [14]. Polymer-modified cementitious coatings have been followed for years in chloride exposure, which gives rare long-term evidence [82].

Hydrophobic treatments have a known weak spot: cracks. Dai et al. showed that the benefit of water-repellent impregnation depends strongly on whether the concrete is cracked [57]. Treatments also age and need to be reapplied. In our view, a surface treatment should be treated in life cycle studies as a maintenance cycle, with its own reapplication interval and its own emissions, not as a one-time addition.

Maturity note. Silane and siloxane impregnations and film-forming coatings are at L5 and TRL 9 (group A). EN 1504-2 covers them [83], and polymer-modified coatings have multi-year monitoring data [82]. Lithium-based and nano-modified coatings are a different case. Their data come from mortar in the laboratory (L2, TRL 4–5, group C) [14].

### 5.2. Coatings on the Steel and Alternative Reinforcement

Steel-level protection includes changes to steel chemistry and coatings on the bar [84]. Epoxy-coated bars work well when the coating is intact, but damage during transport and placing can create local weak spots. Bio-based coatings are being explored. For example, chitosan–gum Arabic nanocomposite coatings on mild steel in saline media raised the water contact angle from about 51° to about 101° and gave good barrier protection [74]. That test was not in concrete, so it shows promise rather than proof. Non-metallic reinforcement, such as glass, basalt, or carbon fiber-reinforced polymer bars, removes electrochemical corrosion altogether but brings new questions on bond, brittleness, and long-term resin degradation [28]. Fibers in the concrete also affect cracking and bond under corrosion [51].

Maturity note. Non-metallic reinforcement has design guides and field use (L4–L5, TRL 8–9). We place it in group B because long-term data in seawater sea-sand concrete are still thin [28]. Bio-based bar coatings have been tested only in liquid media (L1).

### 5.3. Electrochemical Protection and Repair

Electrochemical methods act on the steel directly. Cathodic protection shifts the steel potential so that it no longer corrodes, either with an impressed current or with sacrificial anodes. Electrochemical chloride extraction draws chloride ions out of the cover with a temporary current, and realkalization restores a high pH in carbonated concrete [1,27]. These methods are well proven in the field and can stop active corrosion without removing sound concrete, which saves material and waste. Their sustainability depends on energy use over the years of operation, on the life of the anodes, and on side effects such as changes in pore structure near the steel. Polder et al. pointed out that such non-traditional methods often face non-technical barriers, including owner habits and procurement rules, as much as technical ones [64]. In a life cycle view, a cathodic protection system is a maintenance stream with its own energy and replacement schedule, and it should be modeled that way in Equation (5).

Maturity note. Cathodic protection, chloride extraction, and realkalization are at L5 and TRL 9 (group A), and EN ISO 12696 covers cathodic protection [85]. Their open questions concern energy use and anode life, not whether they work.

## 6. Self-Healing Systems

### 6.1. Why Cracks Matter for Corrosion

Concrete cracks under load, shrinkage, and temperature change. Cracks give chloride, water, and oxygen a short path to the steel [49,57]. Self-healing concrete aims to close these paths without human work. Van Tittelboom and De Belie grouped the approaches into autogenous healing, which relies on continued hydration and calcium carbonate precipitation, and autonomous healing, which uses added agents such as bacteria, capsules, or vascular networks [25]. A later review by De Belie et al. described how self-healing fits into damage management of structures [26].

### 6.2. Bacterial and Biogenic Healing

Jonkers et al. used alkali-resistant, spore-forming bacteria as a healing agent. The bacteria turn a calcium source into calcium carbonate that fills cracks [15]. Achal et al. showed that biogenic treatment improved durability and repaired cracks in concrete [86]. For corrosion, the most useful evidence comes from transport tests. Ling and Qian found that healed cracks in bacterial concrete reduced chloride transmission during electromigration [58]. Bacteria-based healing has also been combined with steel fibers [87]. One caution applies to any living healing agent: microbes can also drive corrosion. Microbiologically influenced corrosion is a major cause of corrosion damage in marine and industrial settings, and its form depends on the microbial environment. Zhang et al. exposed a machined 7075-T6 aluminum alloy to natural seawater with planktonic microbes and to marine sediment with benthic microbes. Seawater produced a continuous corrosion film with marked cracking. Sediment left granular, scattered products that exposed parts of the metal [88]. Aluminum is not rebar, and healing bacteria are selected to precipitate calcium carbonate, not to attack metal [15]. The study does show that the microbial setting shapes the attack. Work on bacterial concrete should therefore check that the bacteria and their metabolites do not change the corrosion conditions at the steel.

### 6.3. Capsule-Based and Nanomaterial-Assisted Healing

Capsule systems release polymers or mineral agents when a crack breaks the capsule. Nanomaterials can support both healing and densification, and their sustainability depends on dosage, energy use in production, and service-life gain [59]. Koleva’s work links self-healing of the matrix with self-healing of the steel surface layer [78]. The concept uses polymeric nanomaterials, mainly polyethylene oxide-b-polystyrene (PEO-b-PS) micelles and vesicles, added at very low doses of about 0.024% by cement mass. In the matrix, the particles seem to promote a more uniform distribution of C-S-H and to reduce pore connectivity. The reported result was about half the porosity and a permeability lower by about three orders of magnitude, and vesicles can also bridge microcracks. At the steel, empty vesicles act mainly as a barrier. Vesicles loaded with calcium hydroxide shifted the open-circuit potential of steel in chloride-containing solution in the anodic direction and raised its polarization resistance. The author linked this to a denser, calcium-rich product layer on the steel. The same additive therefore works on two fronts: it slows chloride transport through the matrix, and it strengthens the protective layer where chloride arrives. The release mechanism is still uncertain. The particles did not respond to pH as first intended, so release by osmotic pressure or by stress from hydration and corrosion products has been proposed instead. Most of the evidence comes from model solutions and mortar, so the dual effect still needs confirmation in reinforced concrete over longer periods.

### 6.4. Critical View

Most self-healing studies measure crack closure, water flow, or strength recovery. Fewer measure chloride profiles at the steel depth or corrosion current. Visual closure at the surface does not prove that the crack is sealed at the bar. We recommend that future work report at least one transport measure at the steel depth and one electrochemical measure, such as corrosion potential or polarization resistance, on healed and unhealed specimens. Without this, we cannot put self-healing into a service-life model.

The gap between crack closure and corrosion protection needs a closer look. First, healing products often form near the crack mouth, where water and CO_2_ are available. A crack can look closed at the surface while it stays open at the depth of the bar. Second, chloride and CO_2_ can enter an open crack soon after exposure, while healing usually takes weeks [25,26]. Corrosion may already have started at the crack tip before the crack seals. Third, sealing a crack does not remove chloride that has already reached the steel, and it does not restore the pH of concrete that has carbonated along the crack faces. In these cases, healing slows further damage but does not return the steel to a passive state. Fourth, healing works best for narrow cracks and needs water. Wider cracks, or cracks in members that stay dry, may not heal at all. Transport tests through healed cracks, such as the electromigration tests of Ling and Qian [58], are a step forward. The next step is to track the corrosion state of bars that cross healed and unhealed cracks over time, because that is what the owner cares about [49,57].

Maturity note. Bacterial healing has reached laboratory and pilot use (L3–L4, TRL 5–6, group B). Capsule and vascular systems have fewer pilots (L2–L4, TRL 4–6, groups B to C). For both, the corrosion benefit rests mainly on laboratory transport tests, such as the electromigration tests in [58].

## 7. Nanomaterials

Nano-silica, nano-titania, nano-clay, carbon nanotubes, and graphene-based materials have been added to cement-based materials to refine pores, act as nucleation sites for hydration products, and block ion transport [28,59]. Nano-silica and carbon nanotubes have also been used to improve seawater sea-sand concrete, which is important for island construction where fresh water and river sand are scarce [28]. Nanoparticles can also serve as inhibitor carriers or as active agents, as in the Elaeis guineensis and silver nanoparticle system [68].

Three problems slow adoption. First, nanoparticles clump together, so mixing energy and dispersants matter as much as dosage. Second, results vary a lot between studies because of differences in particle type, surface treatment, and test method. Third, the production of some nanomaterials is energy-intensive, and their health and environmental effects are not fully known. A nanomaterial that cuts chloride diffusion by a large fraction may still be a poor choice if its production footprint cancels the service-life gain. This is exactly why we argue for life cycle evaluation per year of service life (Section 11).

Practical use raises further questions. Good dispersion in the laboratory often relies on ultrasonication or high-shear mixing, which a ready-mix plant does not have. Colloidal or pre-dispersed products ease this problem, but they add water and cost. The price per kilogram of most nanomaterials is far higher than that of cement, so even a small dose can dominate the material cost of a mix. Reproducibility is a further concern. Reported gains for the same nanomaterial differ widely between studies, and many papers do not report the dispersion state, the particle size in the mix, or the number of batches tested [28,59]. In our view, a nanomaterial result should not be taken as reproducible until it has been repeated with a stated dispersion protocol and an independent material supply. We found few pilot or field applications of nanomaterials aimed at corrosion protection. Handling dry nanopowders on site also raises worker-exposure questions that current guidance does not settle.

Maturity note. Nanomaterial admixtures for corrosion protection are at L2 and TRL 3–5 (group C). The large effects reported for them, such as the porosity and permeability changes in Table 7, come from mortar and model solutions.

## 8. Smart Coatings

Smart or active coatings do more than block the environment. They sense a change and respond. The main triggers are pH, redox potential, chloride, and mechanical damage [89,90]. Shchukin and Möhwald described coatings with nanoreservoirs that release inhibitors when corrosion begins [60]. Vimalanandan et al. built a redox-responsive coating that releases its inhibitor when the potential at a defect shifts, which gives protection only where and when it is needed [61]. Liu et al. reviewed self-healing coatings based on micro- and nanocarriers and discussed how loading capacity and release rate can be controlled [16].

Most smart coating research targets steel in air or water, not steel inside concrete. Transfer to concrete brings new conditions: very high pH, calcium-rich pore solution, abrasion during casting, and the need for good bond. We see two realistic paths. One is smart coatings on the bar with carriers stable at pH 13. The other is cementitious or hybrid surface coatings that carry encapsulated inhibitors near the concrete surface. Both need long-term tests in real concrete before they can enter design.

Maturity note. Smart coatings for steel in concrete are at L1–L2 and TRL 2–4 (group C). Active coatings are well developed for metals in air or water [89,90]. For steel in concrete, none of the studies we found reported a service-life input (Table 7).

## 9. Supplementary Cementitious Materials and Low-Carbon Binders

### 9.1. How SCMs Affect Corrosion

SCMs such as fly ash, ground granulated blast-furnace slag, silica fume, metakaolin, and calcined clay replace part of the clinker. They lower the carbon footprint and, in most cases, improve resistance to chloride [44,91,92]. They work in three ways. They refine the pore structure and so lower the diffusion coefficient. Alumina-rich SCMs bind more chloride. They raise the electrical resistivity of concrete, which slows corrosion once it starts [44,93]. Abd El-Fattah et al. confirmed that the chloride binding capacity of concrete changes with the type of cementitious system [35].

The same reactions have a cost. Pozzolanic reactions consume calcium hydroxide, which lowers the alkaline reserve and can speed up carbonation [9,44]. This means that an SCM that is excellent in a marine splash zone may be weak in a dry, carbonation-dominated setting. Huang et al. summarized practical replacement ranges of about 5% to 15% for silica fume, 5% to 10% for metakaolin, and 20% to 30% for fly ash, while noting that no single SCM is best for all exposures [28]. Table 3 compares the main binder options.

### 9.2. Limestone Calcined Clay Cement

Limestone calcined clay cement (LC3) combines clinker, calcined clay, and limestone. It can cut clinker content by about half while giving similar strength [45,94]. Its dense pore structure and high alumina content give strong resistance to chloride. Pillai et al. combined service-life estimates and LCA for LC3 concrete. They reported that LC3 mixes can be designed to reach annual emissions of about 1.6 to 5 kg CO_2_-eq per cubic meter per year of service life, similar to fly ash concrete and much lower than plain Portland cement concrete in their case [10]. The carbonation resistance of LC3 is lower, so cover and curing matter more.

### 9.3. Alkali-Activated Binders and Emerging SCMs

Alkali-activated materials and geopolymers use industrial by-products with little or no clinker [54]. Adding slag to fly ash-based geopolymer concrete improved its durability indicators in several studies [95,96], and sea sand has been tested in fly ash geopolymer mortars [97]. The corrosion of steel in these binders is less understood than in Portland systems. Pore solution chemistry, sulfide content in slag systems, and carbonation behavior all differ. Standards and long-term field data are also limited. Snellings et al. reviewed new SCM sources, such as calcined clays of lower grade and recycled concrete fines, which will matter as fly ash and slag supplies shrink [55]. Aggressive water exposures, including acid and sulfate, add further limits for all of these binders [98]. Alkaline industrial residues are another possible source. Red mud, the waste from alumina production, is strongly alkaline, and carbonating it can store CO_2_. Fang et al. calcined red mud before carbonation. Calcination made the main minerals more reactive and left a porous, honeycomb-like surface. Under their best conditions (liquid-to-solid ratio 0.3, 20% CO_2_, 0.4 MPa, 30 °C, 1 h), carbon content rose by 23.92%, and their LCA ranked calcination followed by carbonation as the best disposal route [99]. For reinforced concrete, this gain has a drawback. Carbonation lowers alkalinity, and alkalinity is what keeps the steel passive (Section 3.3). A pre-carbonated residue used as an SCM would need checks of pore solution pH and steel passivity before it goes into reinforced members.

Maturity note. The binder options differ widely in maturity. Fly ash, slag, and silica fume have decades of field use and appear in cement and concrete standards (L5, TRL 9, group A). LC3 is in commercial production and in field structures, but long-term corrosion data are limited (L4, TRL 7–8, group B). Alkali-activated binders have laboratory and some field use, few standards, and limited data on steel passivity (L3–L4, TRL 6–7, group B). Pre-carbonated residues such as red mud are still at the laboratory stage for reinforced concrete.

### 9.4. Optimizing the Binder for Performance and Footprint

Choosing a binder is now a multi-objective problem. Hafez et al. built a data-driven tool that weighs functional, economic, and environmental properties of blended cement concrete [100]. Moro et al. proposed a performance-based concept that selects sustainable concretes for chloride exposure by checking both the required service life and the global warming potential [11]. Machine learning with Bayesian tuning has been used to predict the strength of eco-friendly concrete with slag and recycled aggregate, which helps screen mixes quickly [101]. We think this kind of joint screening should become routine.

### 9.5. Seawater, Sea Sand, and Local Materials

In many coastal and island regions, fresh water and river sand are scarce. Using seawater and sea sand saves these resources, but it adds chloride to the concrete from day one. With ordinary steel, this is a serious risk, because the initial chloride content C0 in Equation (1) is no longer zero. Two routes are being explored. One is to pair seawater sea-sand concrete with non-metallic reinforcement, which removes the steel corrosion problem but raises questions about bond and long-term resin durability [28]. The other is to use binders that bind more chloride, such as slag-rich systems, or alkali-activated systems in which sea sand has been tested [35,97]. For hot and arid coastal regions, such as the Arabian Gulf, where high temperature, high salinity, and wet–dry cycling occur together, we see this as one of the most practical research areas in the field.

## 10. Durability-Based Design and Service-Life Modeling

### 10.1. Chloride Ingress Models

Most service-life models for chloride exposure use Fick’s second law. For a semi-infinite medium with a constant surface chloride content, the chloride content C at depth x and time t is

C(x,t) = C0 + (Cs − C0) · [1 − erf( x/(2√(D·t)))](1)

where C0 is the initial chloride content, Cs is the surface chloride content, and D is the apparent diffusion coefficient. Corrosion starts when C at the steel depth reaches the threshold Ccrit. Setting C0 = 0 and solving for the initiation time ti gives

ti = x^2^/(4 · D · z^2^), with z = erf^−1^(1 − Ccrit/Cs) (2)

Khan et al. reviewed these solutions and noted that the Fick-based solution tends to be conservative compared with more detailed models [18]. The water-to-cement ratio changes several inputs at once, including D, Ccrit, tensile strength, and corrosion rate [102]. Temperature and relative humidity also change chloride transport and so the predicted service life [41]. Load-induced damage matters too; bending load changes the diffusion coefficient of steel fiber-reinforced concrete [103]. Sustained tension also matters; Yang et al. studied the tensile creep of ultra-high-performance concrete (UHPC). Creep moved from linear to nonlinear behavior as the stress level rose, and the authors tied this change to a shift in where creep takes place, with the interfacial transition zone and microcracks playing a larger role [104]. Microcracks of this size are hard to detect on site, yet they may raise the effective D of the cover [103]. A diffusion coefficient measured on unloaded specimens may therefore understate transport in members under sustained tension.

### 10.2. Illustrative Calculation

**Figure 3:**
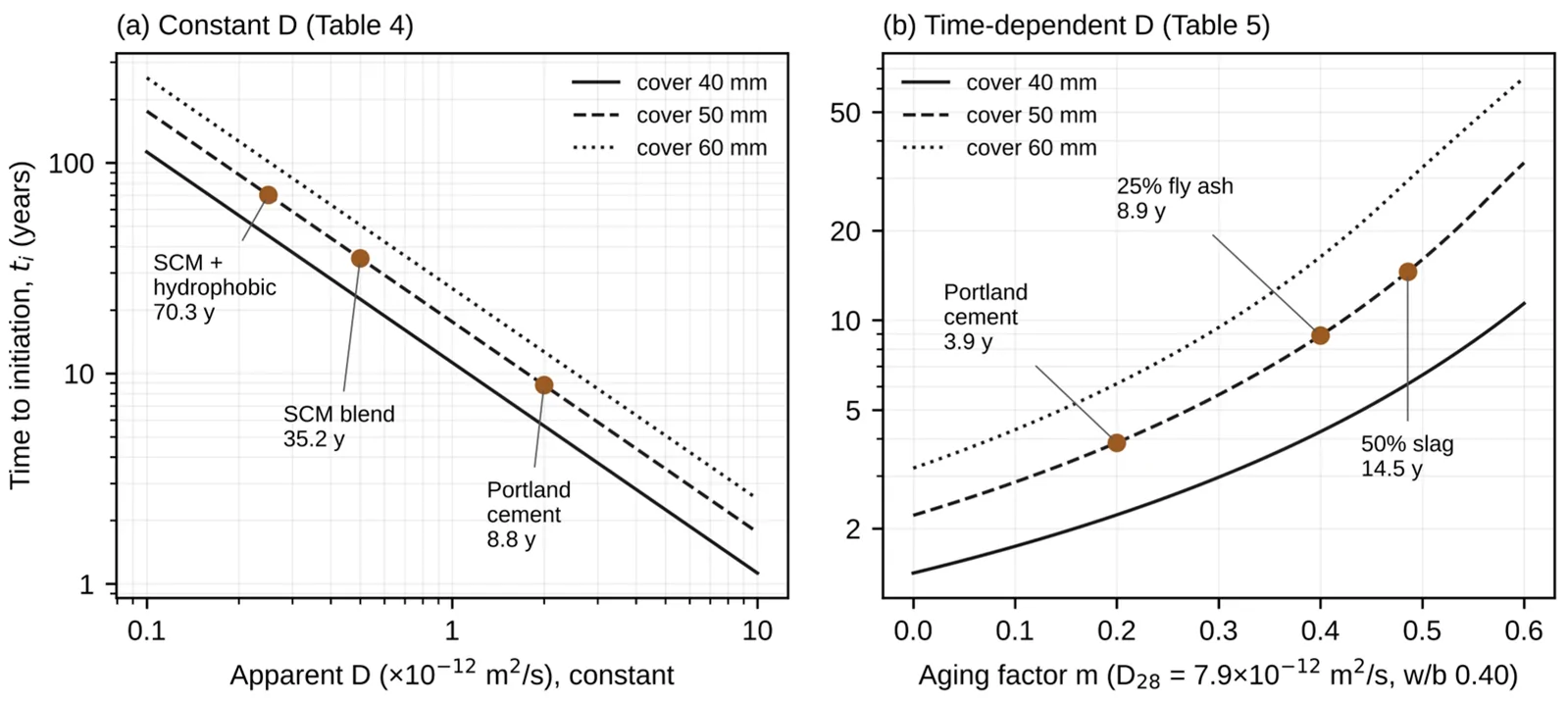
Illustrative time to corrosion initiation (Cs = 3.0%, Ccrit = 0.4% by binder mass, C0 = 0). (**a**) Constant effective D, from Equation (2), for three cover depths; points mark the cases in Table 4. (**b**) Time-dependent D, from Equations (3) and (4), as a function of the aging factor m, with D_28_ = 7.9 × 10^−12^ m^2^/s (w/b = 0.40) and D held constant after 25 years; points mark the Portland cement, 25% fly ash, and 50% slag cases in Table 5. Values are for illustration only.

To show how protection methods enter Equation (2), we ran a simple calculation. The values are illustrative, not measured data. We assumed Cs = 3.0% and Ccrit = 0.4% by binder mass, C0 = 0, a cover of 50 mm, and a constant effective D. Section 10.3 relaxes the constant-D assumption. Step 1: Ccrit/Cs = 0.4/3.0 = 0.133, so erf(z) = 0.867 and z = 1.061. Step 2: 4z^2^ = 4 × 1.127 = 4.51. Step 3: for D = 2.0 × 10^−12^ m^2^/s, ti = (0.05)^2^/(4.51 × 2.0 × 10^−12^) = 2.77 × 10^8^ s, or about 8.8 years. Table 4 and Figure 3 show the same calculation for a binder with a lower D and for that binder with a hydrophobic surface treatment, which we modeled as halving the effective D. This is a conservative choice next to the roughly 75% reduction in apparent diffusion coefficient reported by Coppola et al. [13] in a short test, but it is optimistic over a full service life unless the treatment is renewed (see the limits listed in Section 10.3).

The calculation shows why initiation-phase measures are so powerful. Initiation time scales with the square of the cover and inversely with D. Cutting D by a factor of four through the binder choice raises ti by a factor of four. Adding a treatment that halves D again doubles it. Real structures are less tidy. D falls with age, Cs builds up over time, and Ccrit is uncertain. Still, the example makes clear that the protection method and the design model are not separate topics.

### 10.3. Time-Dependent Diffusivity and the Aging Factor

A constant D is a strong simplification, and it is unfair to SCM concretes. Pozzolanic and latent hydraulic reactions continue for years, so the diffusion coefficient of fly ash and slag concretes keeps falling long after 28 days. Service-life models usually describe this with a power law [40,105]:

D(t) = D_ref_·(t_ref_/t)^m^ (3)

where D_ref_ is the diffusion coefficient measured at the reference age t_ref_ (usually 28 days) and m is the aging factor. D changes with time but not with depth, so Equation (1) still holds if D t is replaced by the integral of D over time. Setting C0 = 0 and solving for the time at which C reaches Ccrit at the steel gives

t_i_ = [x^2^(1 − m)/(4z^2^D_ref_t_ref_^m^)]^1/(1−m)^ (4)

with z defined as in Equation (2). We set the inputs with the relationships of the Life-365 model [105]. For a water-to-binder ratio of 0.40, D_28_ = 10^(−12.06+2.40×0.40)^ = 7.9 × 10^−12^ m^2^/s. The aging factor is m = 0.20 + 0.40 (%FA/50 + %SG/70), with an upper limit of 0.60, where %FA and %SG are the fly ash and slag contents of the binder, respectively. This gives m = 0.20 for Portland cement, 0.40 for 25% fly ash, and 0.49 for 50% slag. As in Life-365, fly ash and slag change m but not D_28_, and D stops falling after 25 years. We kept Cs = 3.0%, Ccrit = 0.4%, and a 50 mm cover, as in Table 4. Table 5 and Figure 3b show the results. The absolute times are short because the full surface chloride content acts from the first day, which matches a severe splash-zone exposure.

**Table 5:** Illustrative time to corrosion initiation with a time-dependent diffusion coefficient, Equations (3) and (4). Inputs: w/b = 0.40, D_28_ from Life-365 [105], t_ref_ = 28 days, D constant after 25 years, Cs = 3.0% and Ccrit = 0.4% by binder mass, C0 = 0, cover 50 mm. Values are for illustration only, not measured data.

#	Case	D_28_ (×10^−12^ m^2^/s)	m	D at 25 Years (×10^−12^ m^2^/s)	t_i_ (Years)	t_i_ Relative to Case 2
1	Portland cement, constant D (m = 0)	7.9	0.00	7.94	2.2	0.6
2	Portland cement	7.9	0.20	2.50	3.9	1.0
3	25% fly ash, constant D (m = 0)	7.9	0.00	7.94	2.2	0.6
4	25% fly ash	7.9	0.40	0.78	8.9	2.3
5	50% slag	7.9	0.49	0.48	14.5	3.7
6	25% fly ash + hydrophobic treatment (D halved)	4.0	0.40	0.39	28.1	7.3

Three results stand out. First, with a constant 28-day D, the fly ash concrete shows no benefit at all over Portland cement, because Life-365 gives both mixes the same D_28_. Once aging is included, the fly ash mix reaches 8.9 years against 3.9 years for Portland cement, and the slag mix reaches 14.5 years. A constant-D calculation based on early-age data would therefore understate the initiation time of these SCM concretes by a factor of about 4 to 7 (rows 3 to 5). Second, the D values at 25 years for the SCM mixes, 0.5 to 0.8 × 10^−12^ m^2^/s, sit close to the effective value we used for the SCM blend in Table 4. The constant D values in Table 4 are therefore best read as long-term effective values; they should not be confused with 28-day test results. Third, the result is very sensitive to m. For the fly ash mix, m = 0.30 and m = 0.50 give 5.6 and 16.0 years. Yet m is rarely measured, and most designers take it from generic tables. Long-term chloride profiles that allow m to be fitted for new SCMs, LC3, and alkali-activated binders are worth as much as better 28-day tests.

These calculations have a narrow range of validity. They assume one-dimensional diffusion into uncracked, water-saturated concrete, a surface chloride content that is reached at once and then stays constant, linear chloride binding, a constant temperature, and a single fixed Ccrit. They fit best for permanently submerged concrete and for the cover zone below the convection zone in tidal and splash areas. They do not hold in the convection zone itself, where capillary suction dominates; in cracked concrete; under combined carbonation and chloride attack; or where temperature varies strongly, as in hot coastal climates [28,41]. The hydrophobic treatment case is the weakest. A silane works mainly by cutting capillary suction, so its real effect is on the convection zone and on Cs, not on D in saturated concrete. Its effect also fades as the treatment ages. Finally, t_i_ covers initiation only. The numbers in Table 4 and Table 5 show trends and the relative weight of each input. They are not design values. Real design needs the probabilistic approach in Section 10.4, with inputs measured on the actual mix.

### 10.4. Probabilistic and Performance-Based Design

Deterministic calculations hide uncertainty. Probabilistic design treats D, Cs, Ccrit, and cover as random variables and reports the probability that corrosion has started by a given time. Leng et al. added the maximum phenomenon, meaning that the first corrosion point along a long member governs, to a time-variant probabilistic model for marine structures [106]. Angst critically examined how well current models predict the time to corrosion initiation and argued that many of their assumptions, especially a fixed Ccrit, are weak [17]. Angst and co-authors listed present and future durability challenges, including the move toward performance-based rules [107]. Earlier research groups built linked models of corrosion that span transport, electrochemistry, and structural response [108].

For the propagation phase, models need a corrosion rate. Otieno et al. showed that rate models are still weak and that field rates vary widely [48]. The work of Sánchez Montero et al. on changing corrosion rate during carbonation-induced propagation shows that a constant rate can mislead [42]. We therefore suggest that designers treat the propagation phase as a reserve, not as a planned part of the service life, unless monitoring is in place.

## 11. Life Cycle Assessment and Life-Cycle Thinking

LCA of concrete often uses 1 m^3^ of concrete as the functional unit. This is simple, but it ignores service life. A better unit for durability studies is the emission per year of service life, which we can write as

GWP_annual = [GWP_initial + ΣGWP_maintenance,i]/t_SL(5)

where GWP_initial is the global warming potential of the materials and construction, GWP_maintenance,i is the footprint of each repair or reapplication, and t_SL is the service life. Pillai et al. used this idea for LC3 concrete and showed that ranking changes once service life is included [10]. Moro et al. applied a similar performance-based logic to chloride exposure [11]. Huang et al. reported that life cycle cost analyses from selected case studies suggest fiber-reinforced polymer bars in seawater sea-sand concrete may cut total life cycle cost by about 30% over 100 years. They also stressed that this depends on maintenance frequency, exposure, discount rate, and material prices [28].

Three points need care. First, maintenance scenarios must be realistic, including reapplication of surface treatments and inhibitors. Second, the service life in the denominator should come from a probabilistic model, and its uncertainty should flow into the LCA. Third, non-traditional assessment and maintenance methods can extend the life of aging structures, and they should be part of the comparison [64]. Angst has also warned that low-carbon binders only help if they do not shorten service life through faster corrosion [9]. We fully agree. A per-year metric with uncertainty bounds is, in our view, the fairest way to compare a green inhibitor, a surface treatment, an SCM, and a non-metallic bar on the same scale.

One more issue affects SCM-based designs. Fly ash and slag are by-products of other industries, and LCA practitioners disagree on how much of the upstream impact should be allocated to them. The choice can change the ranking of blended concretes. We suggest that durability studies report results with and without economic allocation, so that readers can see how sensitive the conclusion is. As supplies of fly ash and slag fall, emerging SCMs such as calcined clays will make this issue less important, but it has not gone away yet [55,100].

We should be clear about what this metric covers. In this review, sustainability is judged mainly by GWP per year of service life. That choice keeps the comparison simple and ties it to the service-life model, but it leaves out other impact categories that matter for some methods. Examples are the ecotoxicity of nitrite and some organic inhibitors, the land and water use behind plant extracts, the energy and health burden of nanomaterial production, and the depletion of scarce SCM sources. Cost and social effects, such as traffic disruption during repair, also fall outside the metric, although they often drive owner decisions [64]. A full sustainability assessment would add these categories, for example through multi-criteria analysis [100]. Our conclusions on sustainability should therefore be read as conclusions on climate impact per year of service.

## 12. Predictive Modeling, Monitoring, and Multi-Scale Evaluation

### 12.1. Machine Learning for Chloride Transport and Corrosion

Machine learning (ML) is now widely used to predict chloride diffusion and corrosion from mix design and exposure data. Table 6 summarizes representative studies. Al Fuhaid and Alanazi compared adaptive neuro-fuzzy inference, artificial neural networks, support vector machines, and extreme learning machines for concrete with SCMs. The support vector model performed best, with R^2^ of 0.955 in training and 0.951 in testing [19]. Hosseinzadeh et al. cleaned a dataset of 1073 experimental results. Extreme gradient boosting (XGBoost) reached R^2^ = 0.94 and support vector regression reached R^2^ = 0.91 for the chloride migration coefficient. They also built classification models and a web tool [20]. Liu et al. optimized several models with particle swarm optimization to predict the chloride diffusion coefficient of silica fume concrete and analyzed feature importance [21].

ML has also been used for corrosion itself. Ahmad et al. trained artificial neural networks on 72 data points with sodium chloride content (0% to 4%), inhibitor dosage (0% to 5%), and exposure time as inputs. The feedforward network reached a correlation coefficient of 0.998 in testing, but the mean absolute percentage error was about 30% [109]. That pair of numbers shows why a single fit metric is not enough. Song et al. used interpretable ML to predict maximum corrosion depth and rank influence factors [110]. Their data came from a buried steel pipeline, so the method may transfer to concrete, but the trained model does not. Bayesian-tuned XGBoost has been applied to eco-friendly concrete with slag [101].

We see three recurring weaknesses. First, datasets are small and drawn from many labs with different test methods, so the models can learn the lab rather than the material. Second, few models respect physical limits, such as the fact that D should not rise when the water-to-binder ratio falls. Third, very few report uncertainty or test on truly new data. Physics-informed neural networks, which build Fick’s law into the loss function, and explainable methods such as SHAP are possible answers, although few have been tested on independent data [28]. A very high R^2^ on a random test split should not be read as proof that a model will work on a new structure. Training on physics-based simulations is another step in this direction. Long et al. trained their NARX network on finite element bond–slip results, so the training data obeyed mechanics by construction, and they added an analytical model to explain the trends [52]. A model built this way can still inherit the errors of the simulation, so it needs the same external tests.

Table 6 puts numbers on these concerns. The datasets range from 72 to about 1000 records, and the 72-point set came from a single study. With a 10% test split, its test metrics rest on about seven points, so a single outlier can change the reported correlation a great deal. None of the studies in Table 6 tested its model on an external dataset from another laboratory or structure. Random splits add a further risk. When several specimens of one mix, or several ages of one specimen, appear in the data, a random split places near-duplicates in both the training and the test sets, and the test score then measures memory rather than prediction. The input data also mix test methods. Migration coefficients from rapid tests, apparent diffusion coefficients from immersion, and values at different ages are not the same quantity, yet they are often pooled. One model in Table 6 was trained on data from a buried steel pipeline, not from concrete, which shows how easily a result can be cited outside its domain [110]. We recommend grouped cross-validation by source study, leave-one-laboratory-out tests, a stated domain of applicability, and prediction intervals rather than point estimates. Until a model passes such checks, we would not use it for design decisions.

Maturity note. ML models for chloride transport and corrosion rest on literature data and have no field validation (TRL 3–4, group C).

### 12.2. Monitoring and Non-Destructive Testing

Monitoring closes the loop between prediction and reality. Half-cell potential mapping remains the most common field method, and RILEM guidance explains how to read it [62]. Polarization resistance measurements give the corrosion rate on site, but they need good control of the polarized area [111,112]. Electrical resistivity is a simple indicator of both transport and corrosion risk [93]. Daniyal and Akhtar reviewed these assessment methods and noted that each has limits, so a combination is best [27].

New sensors can stay inside the concrete. Embedded cement-based acoustic emission sensors have been used for both active and passive monitoring of corrosion [113]. Piezoelectric cement sensors have tracked rebar corrosion in concrete [114]. Digital twins can combine these data streams with models and update service-life estimates as new data arrive [63]. Polder et al. discussed technical and non-technical barriers to using such methods for aging structures [64]. In our view, sensor lifetime inside alkaline concrete is still the main practical limit.

Each monitoring method answers a different question. Potential mapping shows where corrosion is likely. Polarization resistance estimates how fast it is going. Resistivity shows how easily ions and current can move. Embedded sensors give a time history at a few points [62,93,111,113]. No single method gives the full picture, so data fusion is needed [27,28]. For sustainable protection, monitoring has a second role. It tells the owner whether a green inhibitor, a surface treatment, or a self-healing system is still working after years of service. This is the field evidence that the literature now lacks.

Maturity note. Half-cell potential mapping, polarization resistance, and resistivity are routine field methods with RILEM guidance (group A) [62,111]. Embedded sensors and digital twins have reached pilot structures (L4, TRL 5–7, group B).

### 12.3. Multi-Scale Evaluation

Corrosion spans many scales. At the molecular scale, simulations explain how inhibitor films form on the passive layer [65]. At the micro scale, the steel–concrete interface controls where corrosion starts [29,31]. At the meso scale, cracks and heterogeneity create local hot spots [49,50]. At the member scale, section loss and bond loss govern capacity [6,8]. At the network scale, asset managers need probabilities and costs [64,106]. Few studies link more than two of these scales. A sustainable protection method should be judged at the scale where its benefit is claimed and at the scale where decisions are made.

Methods from neighboring fields can help fill these missing links. At the atomic scale, Wang et al. showed that boron oxidizes through a reaction at the boron–oxide interface. The reaction forms a stable B_6_O intermediate, and this intermediate itself slows further oxidation [115]. Models of steel in concrete usually treat the passive film as one uniform oxide. Interface-resolved studies of this kind could test whether intermediate layers form on steel at pH 13 and how inhibitors change them. At the pore scale, nuclear magnetic resonance (NMR) and computed tomography (CT) have been combined to link pore structure and crack distribution with strength in hydrogel-stabilized sand [116]. Pore-scale visualization has also been used to study the microscale barrier mechanisms that slow the transport of microplastics in soils modified with bio-based hydrogel [117]. The same imaging tools could show whether a pore blocker or a healing product really closes the transport path at bar depth, the gap noted in Section 6.4. Designing at two scales at once can also pay off. In machining, Chen et al. combined macro-scale geometric optimization of a forming tool with regional micro-scale edge passivation. Passivation here means rounding the cutting edge, not forming a chemical film. Tool life rose by 47.88% [118]. Corrosion protection follows the same logic. Cover depth and binder choice act at the member scale, the steel–concrete interface acts at the micro scale, and the two work best when they are designed together.

## 13. Evidence Base, Quantitative Comparison, and Technology Maturity

### 13.1. Quantitative Comparison of Reported Effects

Table 7 collects the quantitative effects reported for the main methods, together with the test type behind each number. We include only values stated in the cited sources or derived in Section 10. Three patterns emerge. First, the methods with the largest and best-documented effects on transport are the oldest ones: SCMs, silane treatments, and low water-to-binder ratios. Second, most results for newer methods are given as inhibition efficiency, contact angle, or crack closure. These indicators cannot be entered into a service-life model without further tests, and we could not convert them into changes in D, Ccrit, or corrosion rate. Third, very few studies report the footprint of the added material, so the annual GWP of most methods cannot yet be calculated. The gaps in Table 7 are a finding in their own right. They show where the field needs data before it can compare methods on a fair basis.

### 13.2. Evidence Level and Technology Maturity

Table 8 grades each method by the highest evidence level we found in the reviewed sources, by the state of standards, by its behavior under carbonation, and by an indicative technology readiness level (TRL). The TRL values are our own judgment on the usual scale from 1 (basic principles observed) to 9 (proven in routine operation). They summarize the cited evidence and should be read as a guide, not as a formal rating.

The table separates the methods into three groups. The first group is ready for routine design: SCMs in low-permeability concrete with adequate cover, silane impregnation, film-forming coatings, cathodic protection, and calcium nitrite. These have standards and decades of field use, and their open questions concern optimization and LCA, not feasibility. The second group has reached pilot or early commercial use: LC3, alkali-activated binders, migrating inhibitors, bacterial healing, non-metallic reinforcement, and embedded sensors. These need long-term field data and, in several cases, standards before owners will specify them widely. The third group is still at the laboratory stage: green inhibitors, nano-enabled and encapsulated inhibitors, nanomaterial admixtures, smart coatings for steel in concrete, and ML models for design. For this group, a claim of sustainability is still a hypothesis. For practicing engineers and asset owners, the message is simple. The first group can be specified today. The second can be used with monitoring and a fallback plan. The third belongs in trials.

### 13.3. Integrated Synthesis of the Reviewed Methods

Table 1 and Table 7, and 8 each look at one side of the methods. Table 9 puts the four sides that matter most in practice in one place: durability performance, implementation challenges, sustainability aspects, and the degree of practical validation. The last column repeats the evidence level and TRL from Table 8 and adds the readiness group defined in Section 2. We give a number for durability only where a cited source or Section 10 provides one. Where we write “not reported”, we found no value that a designer could use.

Three points stand out when the columns are read together. First, every method in group A has a documented durability effect and either a standard or a long field record. No method in group C has reported a change in D, Ccrit, or corrosion rate in concrete in a form that Equation (2) can use. Second, the sustainability column is mostly qualitative. Among the methods in Table 9, only LC3 has a published GWP per year of service life in the sources we reviewed [10]. For most other methods, the footprint of the added material or the reapplication interval is missing. Third, the main barriers for group B are long-term field data and standards. Field trials with monitoring would therefore move these methods forward faster than more short laboratory studies.

### 13.4. Cross-Cutting Observations

Reading across Section 4, Section 5, Section 6, Section 7, Section 8, Section 9, Section 10, Section 11 and Section 12, we draw four conclusions of our own. First, novelty and evidence run in opposite directions. The newest methods attract most of the recent papers but have the weakest field evidence, while the methods that protect most structures today receive little new research. Second, a large share of positive results comes from test conditions that favor the method: pore solution instead of concrete, uncracked instead of cracked specimens, and weeks instead of years. We found few negative or null results, which suggests some publication bias. Third, several claimed benefits conflict once a second exposure is considered. SCMs lower chloride diffusion but can speed carbonation and, in some studies, lower the chloride threshold [3,9]. Hydrophobic treatments cut chloride uptake but lose their effect at cracks and do not stop carbonation [1,57]. A method should be called beneficial only for the exposure in which it was tested. Fourth, the sustainability claim of an added material is rarely tested. Few of the studies we reviewed on inhibitors, coatings, healing agents, and nanomaterials reported the production footprint of the added material or the expected life of the protection. These are the two numbers needed to place a method in Equation (5).

## 14. An Integrated Framework for Sustainable Protection

**Figure 4:**
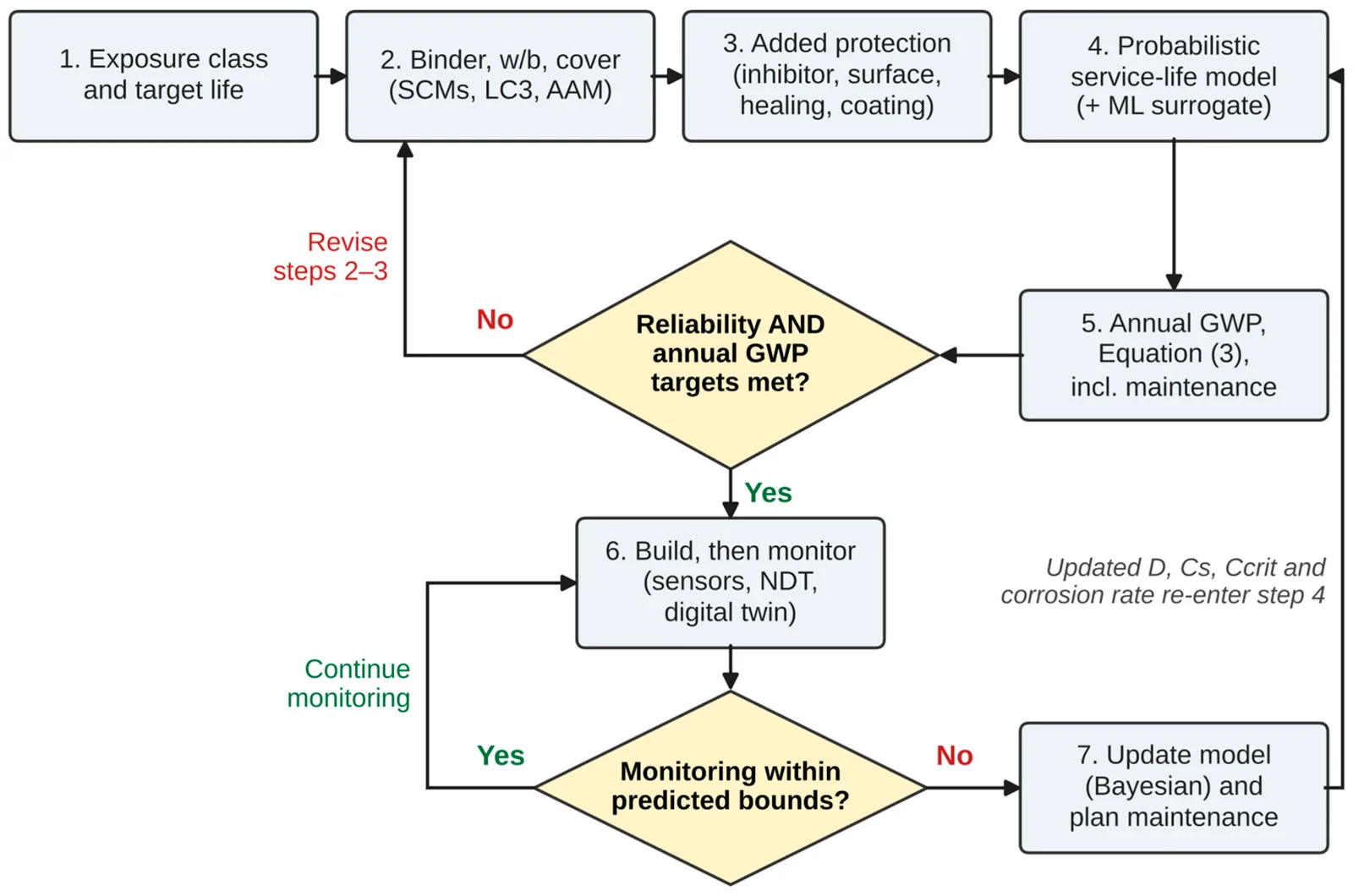
Proposed design loop that links material choice, added protection, probabilistic service-life prediction, life cycle assessment, and monitoring. Diamonds are decision nodes with yes/no branches. The first checks the reliability and annual GWP targets before construction; the second checks monitoring data against the predicted range during service.

Based on this review, we propose the design loop shown in Figure 4. It has seven steps. (1) Define the exposure class and the target service life. (2) Select the binder, water-to-binder ratio, and cover, using SCMs, LC3, or alkali-activated binders where suitable. (3) Add targeted protection only where the model shows it is needed, such as a surface treatment in splash zones or an inhibitor at joints. (4) Run a probabilistic service-life model, with ML surrogates where they are validated. (5) Compute the annual GWP from Equation (5), including planned maintenance. (6) Build and monitor with sensors, non-destructive testing, and, where justified, a digital twin. (7) Update the model with monitoring data and plan maintenance. After step 5, a decision gate checks two targets at once: the reliability target for corrosion initiation at the design service life, and an annual GWP target set by the owner or by a benchmark mix. If either target is missed, the designer returns to steps 2 and 3. After construction, a second gate compares monitoring data with the predicted range. If the data fall outside it, the model is updated and the maintenance plan is revised (step 7), and the updated inputs re-enter step 4.

The loop is not new in its parts. Probabilistic service-life design is codified in the fib Model Code [40], service-life-based LCA has been shown for LC3 and for chloride-exposed concretes [10,11], and digital twins are being developed for monitoring [63]. What we think is new is how the parts are joined. Three features set the loop apart. First, it has a dual gate. A design passes only if it meets the reliability target and the annual GWP target together, so a durable but carbon-heavy design fails as surely as a low-carbon design that corrodes early. Service-life codes check only the first target, and most LCA studies check only the second. Second, it has an evidence filter. A protection method can enter step 3 only with parameters measured with embedded steel in mortar or concrete (evidence level L2 or higher, Section 2), such as a change in D, m, Ccrit, or corrosion rate, together with the expected life of the treatment. Pore-solution data alone do not qualify. Third, maintenance is part of the loop. Monitoring updates the service-life model, the update changes the planned maintenance, and the maintenance emissions feed back into the annual GWP. The sustainability rating of a structure can therefore change during its life. These rules force green inhibitors, coatings, and self-healing agents to report the data that designers need.

Table 10 compares the loop with established frameworks. Each of them covers part of the problem well. The fib Model Code gives a reliability-based check of corrosion initiation [40]. Life-365 adds the aging of D and a life cycle cost [105]. EN 1504-2 sets performance requirements for surface protection products [83]. Pillai et al. and Moro et al. tie the service life to GWP [10,11], and Moro et al. already check service life and GWP together when they select a concrete. Digital twin approaches update models with monitoring data [63]. In the cited sources, we did not find a framework that covers three things at once: a combined check of reliability and GWP per year of service, a rule on what evidence a new protection method needs before it gets credit in design, and the feedback of monitoring into both the maintenance plan and the GWP rating. These three rules are the contribution of the loop.

The rules have practical effects, and three examples use results already given in this review. First, the loop stops a sound low-carbon binder from being rejected for the wrong reason. With a constant 28-day D, the 25% fly ash mix in Table 5 shows no gain over Portland cement, at 2.2 years for both. A designer who trusted that number might add a surface treatment and take on its reapplication emissions. Once the aging factor is measured, as Table 11 asks, the same mix reaches 8.9 years and the slag mix 14.5 years, so the binder does part of the job by itself.

Second, the evidence filter tells the designer which methods can be credited in step 3 today. In Table 7, only SCMs and silane treatments report a change in a transport property that Equation (2) can use. A green inhibitor or a smart coating can still be tried, but it enters the design as a monitored trial, and the structure must meet both targets without it. This protects the owner from paying, in money and in carbon, for a benefit that has not been measured in concrete.

Third, the loop treats maintenance as a stream of emissions that monitoring can change. Take a silane treatment with an assumed ten-year reapplication interval. Over a 50-year life it adds four maintenance terms to Equation (5). If monitoring shows that the cover is still dry at year ten, the interval can be extended and the annual GWP falls. If it shows early wetting, the interval shortens and the rating is revised. A one-time LCA at the design stage cannot capture either case.

Closed-loop control of this kind is already in use elsewhere in structural engineering. Zhang and Li used an active rotary inertia driver (ARID) to damp the swing and torsional vibration of suspended arch rib segments during erection, and they confirmed their model with shake table tests [119]. The time scales differ greatly: seconds for vibration, decades for corrosion. The logic is the same. Measure the response, compare it with a model, and act when the two diverge. The second gate in Figure 4 applies this logic to corrosion management.

To make this practical, Table 11 proposes a minimum reporting checklist for papers on new protection methods. None of the items is hard to measure, but together they would let a designer place the method into Equations (2) and (5) without guessing.

## 15. Research Gaps and Future Directions

Table 12 lists the main gaps we found, ranked by how much they block practical use. The first three gaps are, in our view, the most urgent.

Several further directions deserve attention. Climate change will raise temperatures and change moisture and carbon dioxide levels, which will speed up both carbonation and chloride transport [28,41]. Service-life models should include climate scenarios. Seawater sea-sand concrete with non-metallic bars may help island and coastal construction, but it needs long-term bond and durability data [28,97]. Open, well-documented datasets on chloride transport, thresholds, and corrosion rates would help the ML community far more than new algorithms. Finally, collaboration between corrosion scientists, materials engineers, structural engineers, and LCA practitioners is still rare. The special issue that this review is written for is a good place to build that bridge.

## 16. Conclusions

This review examined sustainable approaches to corrosion protection in reinforced concrete and linked them to design, life cycle, and modeling tools. The main conclusions are as follows.

(1) Corrosion protection and sustainability cannot be judged apart. A method is only green if it lowers emissions per year of service life, including maintenance. Service-life-normalized LCA, as shown for LC3 concrete, should become standard.
(2) Green inhibitors from plant and waste sources show good results in concrete for several extracts, such as bamboo leaf, olive leaf, maize gluten meal, and neem. Most evidence is still short-term. Results from acid solutions should not be used to claim performance in concrete. Their composition varies between batches, and their effect on setting time is rarely quantified.
(3) Among the surface methods we reviewed, silane-based treatments have the most quantitative evidence, with reported cuts in chloride transport of about 30% to 75% depending on the test [13]. Their benefit drops in cracked concrete, and reapplication must be counted in LCA.
(4) Self-healing, nanomaterials, and smart coatings have shown benefits mainly in laboratory tests (evidence levels L1 to L3, with a few bacterial and capsule pilots). They are not yet ready for design. They need tests that report chloride at the steel depth and electrochemical measures, not only crack closure or strength. Closure of a visible crack does not prove that the steel is protected.
(5) In the studies we reviewed, SCMs and LC3 gave the most consistent reductions in chloride diffusion relative to the carbon they save, and they have the longest field record. This ranking comes from a qualitative synthesis of studies with different mixes and test methods, not from a meta-analysis. Their lower alkaline reserve means carbonation must be checked for each exposure, and their benefit is understated when D is treated as constant (Section 10.3).
(6) The chloride threshold is one of the largest uncertainties in service-life prediction. Reported values span more than two orders of magnitude, and in our example a change from 0.2% to 1.0% by binder mass changed the predicted initiation time from about 24 to about 85 years. Better threshold data would improve every other part of the design loop.
(7) Machine learning models predict chloride transport well within their data. They need physics constraints, uncertainty estimates, and tests on independent data before they guide design. None of the ML studies we examined tested its model on an independent external dataset. Monitoring and digital twins can keep these models honest over the life of a structure.
(8) Few of the newer methods have passed the laboratory stage (Table 8). Engineers can specify SCMs, silane treatments, film-forming coatings, and cathodic protection today. Most other methods need pilot use with monitoring before they enter routine design. Table 9 brings durability performance, implementation challenges, sustainability aspects, and practical validation together for each method.
(9) The proposed design loop does not invent new tools. Its contribution is to join existing ones under three rules: a dual gate on reliability and annual GWP, an evidence filter that credits only methods with measured inputs in mortar or concrete, and in-service updating that lets monitoring change both the maintenance plan and the GWP rating (Table 10).

We hope the proposed design loop helps researchers report the data that designers need, and helps designers choose protection methods that make both technical and environmental sense.

## Figures and Tables

**Table 1:** Main families of sustainable corrosion-protection methods, the corrosion stage they act on, and their main limits. Limits are stated as precisely as the cited evidence allows. Table 8 grades the evidence behind each method. Table 9 gives an integrated synthesis of durability, implementation, sustainability, and practical validation.

Method	Main Mechanism	Stage	Sustainability Benefit	Main Limits [Refs]
SCMs, LC3, alkali-activated binders	Pore refinement, chloride binding, higher resistivity	Initiation, and slows propagation	Less clinker; longer initiation	Lower alkaline reserve speeds carbonation (Table 3); slow early strength calls for longer moist curing; fly ash and slag supply is falling [44,45,54,55]
Conventional inhibitors (nitrite, amines, migrating types)	Passive film repair or adsorbed film	Initiation (migrating types: existing structures)	Life extension without demolition	Nitrite needs a minimum nitrite-to-chloride ratio, and under-dosing can intensify pitting; toxicity concerns; for migrating types, there is little proof that enough inhibitor reaches the steel [12,28,47,56]
Green inhibitors (plant or waste extracts)	Adsorption of heteroatom-rich molecules	Initiation	Renewable, low toxicity	Extract composition varies with plant source, season, and extraction method; sugars may retard setting; most data come from short tests in solution [12,22]
Surface treatments (silane, pore blockers, coatings)	Water repellence, pore blocking, barrier film	Initiation	Low material use; suits retrofit	Benefit is lost where cracks cross the treated layer; UV and alkali exposure degrade the agent, so reapplication is needed and adds maintenance emissions [13,24,57]
Self-healing (autogenous, bacterial, capsules)	Crack sealing by carbonate or polymer	Initiation after cracking	Fewer repairs	Crack closure at the surface is no proof of protection at the bar; bacteria and capsules must survive mixing; agents add cost; corrosion benefit rarely measured [15,25,58]
Nanomaterials	Densification, nucleation, inhibitor carriers	Initiation	Effective at small doses	Particles agglomerate unless well dispersed, which is hard at plant scale; results vary between laboratories; energy-intensive production; health data incomplete [28,59]
Smart coatings	Release of inhibitor on pH, redox, or chloride trigger	Initiation and early propagation	Protection on demand	Carriers must stay stable at pH 13 and survive casting; the inhibitor reserve is finite; no long-term data in concrete [16,60,61]
Monitoring and predictive models	Early detection, model updating	Both	Maintenance at the right time	Embedded sensors often fail before the structure ages; potential maps need expert reading; models are only as good as their input data [62,63,64]

**Table 2:** Selected green inhibitors and bio-based coatings reported for steel corrosion protection, grouped by test environment. Class A: steel embedded in concrete under laboratory exposure. Class B: concrete exposed to simulated chloride media. Class C: liquid media without the alkaline cement matrix (acid or neutral saline). Outcomes are summarized as reported by the authors. Results from different classes are not directly comparable, and only Class A and B results should inform concrete design.

Source Material	Test System	Environment Class	Reported Outcome	Ref.
Bambusa arundinacea leaf extract	Steel in reinforced concrete	A	Reduced corrosion; proposed as a sustainable admixed inhibitor	[67]
Elaeis guineensis extract with silver nanoparticles	Reinforced concrete	A	Improved corrosion resistance of embedded steel	[68]
Plant-based eco-addition inhibitor	Steel embedded in concrete	A	Mitigated corrosion of reinforcing steel	[69]
Olive leaf extract	Reinforced concrete contaminated with seawater	A	Acted as a green inhibitor under seawater contamination	[70]
Maize gluten meal extract	Steel in concrete, with theoretical calculations	A	Inhibition mechanism explained by combined experiments and calculations	[71]
Neem (Azadirachta indica) leaf extract	Reinforced concrete in simulated chloride media	B	Compared with three commercial inorganic inhibitors; no change to concrete integrity	[72]
Fenugreek seed and cape gooseberry leaf extracts	Steel in phosphoric acid	C	Effective in acid; not directly transferable to alkaline concrete	[73]
Chitosan–gum Arabic nanocomposite (coating)	Mild steel in saline media	C	Water contact angle rose from about 51° to about 101°; good barrier protection	[74]

**Table 3:** Qualitative comparison of binder options for corrosion protection. Replacement ranges follow [28]; performance depends on mix design, curing, and exposure.

Binder	Chloride Resistance	Carbonation	Clinker/CO_2_ Saving	Notes [Refs]
Fly ash (about 20–30%)	Lower D with age; added binding	Can increase (less portlandite)	Moderate to high	Slow early strength; long curing needed [44,91]
Ground granulated blast-furnace slag	Strong binding (alumina); lower D	Can increase	High	Supply may fall with steel decarbonization [36,44,55]
Silica fume (about 5–15%)	Strong pore refinement	Can increase at high dose	Low (small dose)	Cost; sensitive to curing [28,92]
Metakaolin (about 5–10%)	Binding and refinement	Can increase	Moderate	Higher water demand [28,92]
Limestone calcined clay cement (LC3)	Very low D; good binding	Lower than Portland cement	About half the clinker	Cover and curing matter more [10,45,94]
Alkali-activated and geopolymer binders	Varies; can be good with slag	Often faster	Very high	Limited standards and steel passivity data [54,95,96]

**Table 4:** Illustrative time to corrosion initiation from Equation (2) with Cs = 3.0% and Ccrit = 0.4% by binder mass, C0 = 0, and constant effective D. These D values are best read as long-term effective values, not 28-day test results; Table 5 treats D as time-dependent. Values are for illustration only, not measured data.

Case	D (×10^−12^ m^2^/s)	Cover (mm)	ti (Years)
Portland cement concrete (reference)	2.0	50	8.8
SCM blend	0.5	50	35.2
SCM blend + hydrophobic treatment (D halved)	0.25	50	70.3
SCM blend, reduced cover	0.5	40	22.5
SCM blend, increased cover	0.5	60	50.6

**Table 6:** Representative machine learning studies on chloride transport and corrosion. Metrics are as reported by the authors on their own data splits. The last column states how each model was validated and whether it was tested on data independent of the training source; none of the studies reported such an external test.

Study	Target	Data	Models	Reported Performance	Validation and External Test
Al Fuhaid and Alanazi [19]	Chloride diffusion coefficient, SCM concrete	105 literature data points with C3A, slag, fly ash	ANFIS, ANN, SVM, ELM	SVM best: R^2^ = 0.955 (train), 0.951 (test)	70/30 hold-out with 10-fold cross-validation; no external test set
Hosseinzadeh et al. [20]	Chloride migration coefficient and resistance class	1073 results from 24 studies (965 after outlier removal)	XGBoost, SVM, RF, LightGBM	XGBoost R^2^ = 0.94; SVM R^2^ = 0.91; classification accuracy up to 0.97	80/20 hold-out, SMOTE on training data only; no external test set
Liu et al. [21]	Chloride diffusion coefficient, silica fume concrete	Literature data	BP-ANN, SVR, GP, XGBoost, RF with PSO	Model comparison and feature analysis	Internal validation on pooled literature data; no external test set reported
Ahmad et al. [109]	Corrosion rate of steel in cementitious composites in clay soils	72 data points from one source study; NaCl 0–4%, inhibitor 0–5%, 30–180 days	Feedforward and cascade-forward ANN	Feedforward: R = 0.998, MAPE = 30.43%	Random 80/10/10 split (about 7 test points); no external test set
Song et al. [110]	Maximum corrosion depth	240 field samples from one buried steel pipeline (not concrete)	Interpretable ML	Ranking of influence factors	Random 200/40 split, 10 repeats; no external test set
Al-Taai et al. [101]	Compressive strength of eco-friendly concrete	Slag and recycled aggregate mixes	XGBoost with Bayesian tuning	Screening tool for low-carbon mixes	5-fold cross-validation; no external test set reported

**Table 7:** Quantitative effects reported for selected protection methods, with the test basis and evidence level (L1–L5, Section 2). Values are as reported in the cited studies; they come from different materials and test methods and should not be ranked against each other without regard to the test basis.

Method	Indicator	Reported Change	Test Basis (Evidence Level)	Ref.
Silane-based surface-applied inhibitor	Non-steady-state migration coefficient	30–60% lower than untreated	Accelerated migration test (L2)	[13]
Silane-based surface-applied inhibitor	Apparent chloride diffusion coefficient	About 75% lower than untreated	Natural diffusion test (L2)	[13]
LC3	Clinker content	About 50% lower than Portland cement	Mix design	[45,94]
LC3 concrete	GWP per year of service life	About 1.6–5 kg CO_2_-eq per m^3^ per year; similar to fly ash concrete and much lower than Portland cement concrete in that study	Service-life model with LCA (case study)	[10]
25% fly ash; 50% slag	Time to corrosion initiation	About 2.3 and 3.7 times that of Portland cement when the aging of D is included	Model calculation, Table 5 (illustrative)	This review, [105]
SCM blend + hydrophobic treatment	Time to corrosion initiation	About 8 times that of Portland cement with constant effective D	Model calculation, Table 4 (illustrative)	This review
FRP bars in seawater sea-sand concrete	Life cycle cost over 100 years	About 30% lower; depends on discount rate and maintenance	Life cycle cost analysis (selected case studies)	[28]
PEO-b-PS nanomaterials (about 0.024% of cement mass)	Porosity; permeability	About 50% lower; about three orders of magnitude lower	Mortar and model solution (L1–L2)	[78]
Bacterial self-healing	Chloride transport across healed cracks	Lower than across unhealed cracks	Electromigration test (L2)	[58]
Chitosan–gum Arabic coating	Water contact angle	From about 51° to about 101°	Mild steel in saline solution (L1)	[74]
Green inhibitors (several extracts)	Change in D, Ccrit, or corrosion rate in concrete	Not reported in a form usable in Equation (2)	Mostly L1–L2	[12,67,68,69,70,71,72]
Smart coatings for steel in concrete	Any service-life input	Not reported	L1	[16,60,61]

**Table 8:** Evidence level, standards, behavior under carbonation, and indicative technology readiness level (TRL) of the reviewed protection methods. L1: liquid media; L2: accelerated laboratory tests on mortar or concrete; L3: long-term laboratory or natural exposure; L4: pilot or field application; L5: field performance over ten years or more. TRL values are the authors’ judgment based on the cited evidence.

Method	Highest Evidence Level Found	Standards or Guidance	Effect Under Carbonation	Indicative TRL
SCMs (fly ash, slag, silica fume)	L5: decades of field use	Covered by cement and concrete standards	May speed carbonation; needs low w/b and adequate cover	9
LC3	L4: commercial production and field structures; long-term data limited	Depends on national standards	Lower carbonation resistance; cover and curing are critical	7–8
Alkali-activated binders	L3–L4: laboratory and some field use	Few standards; mostly performance-based acceptance [54]	Often carbonates faster; steel passivity data limited	6–7
Calcium nitrite (admixed)	L5	ASTM C1582 [66]	Little evidence; designed for chloride	9
Migrating inhibitors	L4: field use; proof of arrival at the steel weak	Product-specific	Mixed evidence	6–8
Green inhibitors	L2: laboratory concrete, mostly short tests	None	Not studied in the reviewed sources	3–4
Encapsulated and nano-enabled inhibitors	L1–L2	None	Not studied	2–4
Silane and siloxane impregnation	L5	EN 1504-2 [83]	Does not stop CO_2_; lowers corrosion rate by keeping the cover dry	9
Film-forming coatings, polymer-modified mortars	L5 (multi-year monitoring [82])	EN 1504-2 [83]	Anti-carbonation coatings slow CO_2_ ingress	9
Lithium-based and nano-modified coatings	L2	None	Not studied	4–5
Cathodic protection, chloride extraction, realkalization	L5	EN ISO 12696 [85]	Effective; realkalization is designed for carbonated concrete	9
Bacterial self-healing	L3–L4: laboratory and pilot applications	None	Healing does not restore pH at the bar	5–6
Capsule and vascular healing	L2–L4 (few pilots)	None	Not studied	4–6
Nanomaterial admixtures	L2	None	Densification may slow CO_2_; little data	3–5
Smart coatings for steel in concrete	L1–L2	None	Not studied	2–4
Non-metallic (FRP) reinforcement	L4–L5	Design guides exist	Not relevant (no steel)	8–9
Embedded sensors and digital twins	L4: pilot structures	Limited	Applies to both exposures	5–7
ML models for chloride transport and corrosion	Literature data only; no field validation	None	Trained mostly on chloride data	3–4

**Table 9:** Integrated synthesis of the reviewed protection methods: durability performance, implementation challenges, sustainability aspects, and degree of practical validation. Validation gives the highest evidence level found (L1–L5, Section 2), the indicative TRL (Table 8), and the readiness group: A, ready for routine design; B, pilot or early commercial use, to be used with monitoring; C, laboratory stage, suitable for trials only. Quantified effects are as reported in the cited sources or derived in Section 10; they come from different materials and tests and are not directly comparable across rows.

Method	Durability Performance	Implementation Challenges	Sustainability Aspects	Practical Validation (Level; TRL; Group)
SCMs (fly ash, slag, silica fume)	Lower D, more chloride binding, higher resistivity. With aging of D, initiation time about 2.3–3.7 times that of Portland cement (Table 5, illustrative). May speed carbonation.	Falling fly ash and slag supply; longer moist curing; carbonation check needed for each exposure	Less clinker; allocation of by-product impacts can change the ranking (Section 11)	L5; TRL 9; A
LC3	High chloride resistance from dense pores and alumina; lower carbonation resistance	Clay quality; cover and curing are critical; standards vary by country	About 50% less clinker [45,94]; about 1.6–5 kg CO_2_-eq per m^3^ per year of service in one case study [10]	L4; TRL 7–8; B
Alkali-activated binders	Better durability indicators with added slag [95,96]; steel passivity and carbonation less understood	Few standards; variable precursors; sulfide in slag systems	Little or no clinker; activator production must be counted in LCA	L3–L4; TRL 6–7; B
Calcium nitrite (admixed)	Protects when dosed above the required nitrite-to-chloride ratio	Under-dosing can intensify pitting; dose tied to expected chloride	No change of binder; ecotoxicity lies outside the GWP metric	L5; TRL 9; A
Migrating inhibitors	Applied to existing structures; effect at the steel uncertain	Little proof that enough inhibitor reaches the steel	Avoids demolition; reapplication must be counted	L4; TRL 6–8; B
Green inhibitors	Lower corrosion in short laboratory tests in concrete [67,70,71,72]; no data on D, Ccrit, or corrosion rate	Batch variability; possible set retardation; supply volume	Renewable, low toxicity; land and water use not reported	L2; TRL 3–4; C
Encapsulated and nano-enabled inhibitors	Triggered release shown in the laboratory	Carrier stability at pH 13; cost; effect on hydration	Carrier footprint not reported	L1–L2; TRL 2–4; C
Silane and siloxane impregnation	Apparent D about 75% lower, migration coefficient 30–60% lower [13]; benefit lost at cracks; does not stop CO_2_	Reapplication; crack sensitivity; aging under UV and alkali	Low material use; reapplication emissions	L5; TRL 9; A
Film-forming coatings, polymer-modified mortars	Multi-year protection in chloride exposure [82]; anti-carbonation types slow CO_2_	Surface preparation; aging of the film	Maintenance cycles enter Equation (5)	L5; TRL 9; A
Lithium-based and nano-modified coatings	Salt resistance on mortar [14]; no data with embedded steel	Long-term bond and aging unknown	Footprint not reported	L2; TRL 4–5; C
Cathodic protection, chloride extraction, realkalization	Stops active corrosion; realkalization restores pH	Specialist design; anode life; owner and procurement barriers [64]	Avoids removal of sound concrete; operating energy and anode replacement	L5; TRL 9; A
Bacterial self-healing	Lower chloride transport across healed cracks [58]; does not restore pH at the bar	Survival of bacteria during mixing; needs water; narrow cracks only; cost	Fewer repairs; footprint of healing agent rarely reported	L3–L4; TRL 5–6; B
Capsule and vascular healing	Crack sealing; corrosion effect rarely measured	Capsule survival during mixing; finite healing agent	Footprint of healing agent rarely reported	L2–L4; TRL 4–6; B–C
Nanomaterial admixtures	About 50% lower porosity and about three orders lower permeability in mortar (PEO-b-PS) [78]	Dispersion at plant scale; cost; reproducibility; worker exposure	Energy-intensive production may cancel the gain	L2; TRL 3–5; C
Smart coatings for steel in concrete	No service-life input reported	Stability at pH 13; abrasion during casting; finite inhibitor reserve	Not reported	L1–L2; TRL 2–4; C
Non-metallic (FRP) reinforcement	No electrochemical corrosion; bond and resin aging remain open	Brittle behavior; bond; long-term data in seawater sea-sand concrete	With seawater sea-sand concrete, about 30% lower life cycle cost over 100 years in selected cases [28]; saves fresh water and river sand	L4–L5; TRL 8–9; B
Embedded sensors and digital twins	Early detection of corrosion; model updating	Sensor life shorter than the structure	Maintenance at the right time	L4; TRL 5–7; B
ML models for chloride transport and corrosion	Good fit inside training data; no external test (Table 6)	Small, mixed datasets; few physics constraints or uncertainty estimates	Could reduce testing effort	Literature data only; TRL 3–4; C

**Table 10:** Comparison of the proposed design loop with existing durability-oriented and sustainability-oriented frameworks. Entries describe the scope of each framework as used in the cited source. They are not a full appraisal of each framework. “Not the focus” means the cited source does not treat the item as a main element.

Framework	Main Purpose	Durability Check	Climate Impact	New Protection Methods	Use of Monitoring
fib Model Code for Service Life Design [40]	Probabilistic service-life design	Reliability of corrosion initiation	Not included	Not the focus	Not the focus
Life-365 [105]	Service life and life cycle cost under chloride exposure	Initiation and propagation; aging of D	Not included (cost only)	Built-in options for common protection measures	Not the focus
EN 1504-2 [83]	Requirements for surface protection products	Product performance tests	Not included	Listed product types only	Not the focus
Pillai et al. [10]	Service life and LCA of LC3 concrete	Service-life estimate	GWP per year of service life	Binder choice only	Not included
Moro et al. [11]	Performance-based selection of concrete for chloride exposure	Required service life	GWP checked together with service life	Binder and mix choice	Not the focus
Digital twin approaches [63]	Model updating from monitoring data	Updated condition and service-life estimates	Not the focus	Not the focus	Core feature
This review (Figure 4)	Design and management loop for protected reinforced concrete	Reliability of initiation at the design life (gate 1)	Annual GWP from Equation (5), including maintenance (gate 1)	Credited only with L2 or higher data on D, m, Ccrit, or corrosion rate, plus treatment life	Gate 2 updates the model, the maintenance plan, and the GWP rating

**Table 11:** Proposed minimum reporting checklist for studies on sustainable corrosion-protection methods.

Item	What to Report	Used in
Test medium and duration	Solution, mortar, concrete, or field; exposure length in days or years; evidence level L1–L5 (Section 2)	Judging transfer to practice
Exposure type	Chloride, carbonation, or combined; cracked or uncracked specimens	Judging which exposure the result covers
Transport property	Change in apparent D or migration coefficient versus a reference mix	Equation (2), initiation time
Aging of transport property	Aging factor m from chloride profiles at two or more ages	Equations (3) and (4)
Chloride threshold	Ccrit with and without the method, measured with steel embedded	Equation (2), initiation time
Corrosion rate	Polarization resistance or mass loss after initiation	Propagation phase
Effect on concrete	Setting time, strength, and shrinkage	Structural design checks
Durability of the method	Expected life of the treatment, inhibitor, or coating; reapplication interval	Maintenance terms in Equation (5)
Environmental data	Dose per m^3^ and production footprint of the added material	GWP_initial in Equation (5)
Uncertainty	Number of specimens, scatter, and variability between batches	Probabilistic design

**Table 12:** Main research gaps, ranked by how much they block practical use, with suggested actions.

#	Gap	Why It Matters	Suggested Action
1	Few long-term tests of green inhibitors and smart coatings in real concrete	Short solution tests overstate benefit; designers cannot use them	Multi-year exposure of reinforced specimens; report change in Ccrit and corrosion rate
2	Large uncertainty in the chloride threshold	One of the inputs with the largest effect on predicted initiation time (Section 3.2)	Agreed test methods; report distributions, not single values [3,37]
3	LCA studies that ignore service life and maintenance	Can rank short-lived options as green	Use GWP per year of service life with probabilistic t_SL, Equation (5)
4	Self-healing studies without corrosion measures	Crack closure at the surface does not prove protection at the bar	Measure chloride at bar depth and corrosion potential or rate
5	Unknown interaction between inhibitors and SCMs	Adsorption on hydrates may reduce inhibitor supply	Factorial tests of inhibitor type and binder type
6	Limited steel corrosion data and standards for alkali-activated binders	Blocks use of very low-carbon binders in reinforced members	Pore solution, passivity, and long-term exposure studies
7	Small, mixed ML datasets without physics or uncertainty	High R^2^ does not mean good field predictions	Open datasets, physics-informed models, independent validation
8	Short life of embedded sensors	Monitoring stops before the structure needs it	Durable sensor materials and self-sensing concrete
9	Unknown footprint and safety of nanomaterials	Production may cancel service-life gains	Include nanomaterial production in LCA; health data
10	Climate change not in service-life models	Warmer, wetter, higher-CO_2_ futures speed deterioration	Climate scenarios in probabilistic design [41]
11	Few tests of protection methods under carbonation	Most data are for chloride exposure, and benefits may not transfer	Tests under carbonation and combined exposure; report carbonation depth and corrosion rate
12	Aging factor rarely measured for new binders	Initiation time is very sensitive to m (Section 10.3)	Long-term chloride profiles at several ages for SCM, LC3, and alkali-activated concretes

## Data Availability

No new data were created or analyzed in this study. Data sharing is not applicable to this article. The illustrative calculations in Section 3.2 and Section 10.2, and 10.3 can be reproduced from Equations (1)–(4) with the inputs given in the text and in Table 4 and Table 5.

## Abbreviations

AAM	Alkali-activated material
ANN	Artificial neural network
Ccrit	Critical chloride content (chloride threshold)
Cs	Surface chloride content
D	Apparent chloride diffusion coefficient
GWP	Global warming potential
LC3	Limestone calcined clay cement
LCA	Life cycle assessment
MCI	Migrating corrosion inhibitor
ML	Machine learning
SCM	Supplementary cementitious material
SVM	Support vector machine
XGBoost	Extreme gradient boosting
D28	Diffusion coefficient at 28 days
FA	Fly ash
FRP	Fiber-reinforced polymer
m	Aging factor of the diffusion coefficient
NDT	Non-destructive testing
PEO-b-PS	Polyethylene oxide-b-polystyrene
SG	Ground granulated blast-furnace slag
SHAP	Shapley additive explanations
TRL	Technology readiness level
w/b	Water-to-binder ratio
